# Green Hydrothermal Synthesis of Fluorescent 2,3‐Diarylquinoxalines and Large‐Scale Computational Comparison to Existing Alternatives

**DOI:** 10.1002/cssc.202100433

**Published:** 2021-03-26

**Authors:** Fabián Amaya‐García, Michael Caldera, Anna Koren, Stefan Kubicek, Jörg Menche, Miriam M. Unterlass

**Affiliations:** ^1^ Institute of Applied Synthetic Chemistry Technische Universität Wien Getreidemarkt 9/163 1060 Vienna Austria; ^2^ Institute of Materials Chemistry Technische Universität Wien Getreidemarkt 9/165 1060 Vienna Austria; ^3^ CeMM Research Center for Molecular Medicine of the Austrian Academy of Sciences Lazarettgasse 14 1090 Vienna Austria; ^4^ Max Perutz Labs Campus Vienna Biocenter 5 1030 Vienna Austria

**Keywords:** computational analysis, fluorescence, green chemistry, hydrothermal synthesis, quinoxalines

## Abstract

Here, the hydrothermal synthesis (HTS) of 2,3‐diarylquinoxalines from 1,2‐diketones and *o*‐phenylendiamines (*o*‐PDAs) was achieved. The synthesis is simple, fast, and generates high yields, without requiring any organic solvents, strong acids or toxic catalysts. Reaction times down to <10 min without decrease in yield could be achieved through adding acetic acid as promoter, even for highly apolar biquinoxalines (yield >90 % in all cases). Moreover, it was shown that HTS has high compatibility: (i) hydrochlorides, a standard commercial form of amines, could be used directly as combined amine source and acidic catalyst, and (ii) Boc‐diprotected *o*‐PDA could be directly employed as substrate that underwent HT deprotection. A systematic large‐scale computational comparison of all reported syntheses of the presented quinoxalines from the same starting compounds showed that this method is more environmentally friendly and less toxic than all existing methods and revealed generic synthetic routes for improving reaction yields. Finally, the application of the synthesized compounds as fluorescent dyes for cell staining was explored.

## Graphical abstract

**In hot water**: 2,3‐Diarylquinoxalines and biquinoxalines are synthesized in a rapid, efficient and green fashion in nothing but high‐temperature water. Through a large‐scale computational analysis, the herein reported reaction conditions are compared with all reported syntheses of the presented compounds.

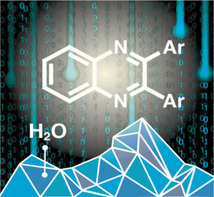

## Introduction

The necessity of implementing and increasing sustainability in all aspects of human life is nowadays widely accepted. For instance, towards this end, the United Nations have in 2015 published the “2030 Agenda for Sustainable Development” including the formulation of 17 “Sustainable Development Goals”.^[1]^ Chemistry is a major cornerstone of modern life, contributing both to the molecular understanding of various natural and synthetic processes, and the design and manufacture of various products, from personal care commodities to pharmaceuticals and materials for devices. Hence, implementing sustainable approaches in chemistry is potentially impactful in numerous aspects of modern life. Conceptualized already in the 1990s, Green Chemistry comprises the ambition to make chemical synthesis and production more sustainable,^[2]^ which is however quite complex: The generation of chemical compounds involves several protagonists (i. e., precursors, the actual transformation of precursors to products, products and side products), and aspects of sustainability/green‐ness are also numerous (e. g., toxicity, hazardousness, renewability, (bio‐)degradability, recyclability) and have to be considered for all involved protagonists. Research towards the improvement of any of these aspects is important in its own right, and advances in all aspects will collectively ultimately lead to fully sustainable chemical synthesis and production. The aspect of the actual transformation from precursors to products is already a major focus in industry, but still lacks sufficient implementation on the research lab‐scale. This is especially true in organic synthesis, which is often intensively employing multi‐step syntheses and purifications, often requiring toxic catalysts and large volumes of petroleum‐derived solvents and auxiliaries. Implementing new synthetic approaches in organic research labs requires fulfilling criteria such as (i) rapidity, (ii) efficiency (small number of reaction steps, high yields, easy product purification), and (iii) versatility (e. g., broad scope of starting compounds). New green approaches are not exempt from fulfilling these criteria, and additionally have to provide (iv) low environmental impact and toxicity. Clearly, developing green synthesis routes is particularly important for abundant compound classes.

**Scheme 1:**
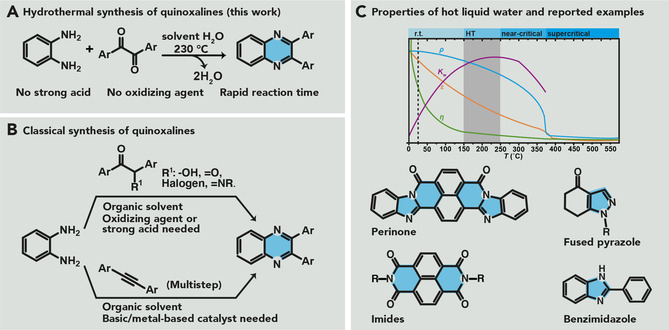
(A) Synthetic approach of this work. (B) Classical synthesis towards 2,3‐diarylquinoxalines. (C) Schematic of the physicochemical properties of water (*ϵ*=static dielectric constant, *η*=viscosity, *ρ*=density, *K*_w_=self‐ionization of water) as a function of temperature (adapted from references [29, 30]) and examples of heteroaromatics obtained between 150–250 °C (in the HT regime).

Here, we have set out to both show that heteroaromatic quinoxalines can be synthesized in nothing but liquid high‐temperature water (HTW), under so‐called hydrothermal (HT) conditions (Scheme 1A), and to demonstrate, for the first time, the broad synthetic applicability of an organic hydrothermal synthesis (HTS) with respect to substrate and product scope, and compatibility with protecting group strategies. The moiety of choice, quinoxalines, also known as 1,4‐benzopyrazines, are a key scaffold among the important nitrogen‐containing heteroaromatic compounds.

Quinoxalines are widely used in medicine, for example, as antibacterial, antifungal, or anticancer agents,[^3^, ^4^, ^5^] and also as building blocks in advanced materials.[^6^, ^7^, ^8^] Various syntheses of quinoxalines have been reported, most employing 1,2‐diamines in combination with different carbonyl compounds (Scheme 1B), that is, mainly diketones or α‐substituted carbonyl compounds (e. g., α‐hydroxyketones,[^9^, ^10^, ^11^, ^12^] α‐bromoketones^[13]^ or α‐oximeketones^[14]^), and the transformations require the presence of oxidizing agents. The vast majority of reported syntheses of quinoxalines use traditional organic solvents and require reaction times (*t*_r_) of several hours. Furthermore, most reported syntheses rely on catalysts, of which a wide variety has been explored.^[15]^ A handful of works on quinoxaline synthesis have targeted part of the aforementioned criteria (i)–(iv) for the implementation of green synthesis. Specifically, reported green approaches focus either on shortening *t*_r_ by using a combination of catalysts and microwave (MW) heating,[^16^, ^17^, ^18^, ^19^, ^20^] or using greener solvents.^[21]^ However, not a single work has to date addressed all criteria. Herein, we have set out to show that all criteria can be simultaneously fulfilled for quinoxalines through HTS.

HTS designates using the reaction medium liquid water above its boiling point but significantly below its critical point, that is, broadly defined at reaction temperatures *T*_r_ of 100–250 °C, and more narrowly defined at approximately 150 °C≤*T*_r_≤250 °C. HTS has been employed to generate inorganic compounds for over 150 years. The technique is geomimetic by imitating the natural mineral formation process “hydrothermal crystallization”, through which various minerals form naturally, including many metal oxides (e. g., SiO_2_, Al_2_O_3_), many silicates (e. g., olivine (Mg, Fe)_2_SiO_4_), and all‐natural zeolites. Interestingly, these minerals are all formed by cyclocondensation reactions with H_2_O as byproduct. At first glance, it seems counterintuitive for a reaction product to form in its byproduct as medium. Yet, research in the field of HTS of minerals has shown that HTW possesses dehydrating abilities favoring (cyclo‐)condensations with H_2_O as byproduct.^[22]^

In recent years, several reports provided evidence of the suitability of HTS to also generate organic (cyclo‐)condensation products. The reported examples to date comprise amides,^[23]^ and in the realm of surprisingly apolar heteroaromatics: cyclic 5‐ and 6‐membered imides fused with aromatic rings,[^24^, ^25^] benzimidazoles,[^26^, ^27^] and perinones.^[28]^ Besides the hydrothermal regime, there are other regimes of liquid HTW: (i) the supercritical regime (*T*_r_>*T*_c_ (374.2 °C) and *p*_r_>*p*_c_ (22.1 MPa), with *T*_r_ and *p*_r_=reaction temperature and pressure, *T*_c_ and *p*_c_=critical temperature and pressure), and (ii) the subcritical or near‐critical regime (250 °C< *T*_r_ < *T*_c_ and 1 bar<*p*_r_<*p*_c_). These regimes vary substantially in physicochemical properties, and consequently also in the necessary equipment. First, the autogenous pressure of water is still moderate until 250 °C, but relatively high at >250 °C, that is, in proximity to the critical point. Hence, synthesis in sub‐ and supercritical water places more stringent demands on reaction vessel engineering than HTS. Second, through structural changes in H_2_O_(l)_ with *T*, properties that are very relevant for synthesis, such as density, viscosity, polarity, and acidity/basicity, also change drastically and with different signatures.[^29^, ^30^, ^31^] The density and viscosity of H_2_O_(l)_ decrease with *T*, which is beneficial for mass transfer in all regimes, often resulting in astonishingly short *t*_r_. Polarity‐wise, the lower HT regime is still quite polar, while the HT regime at 150 °C≤*T*_r_≤250 °C spans relative permittivities of approximately 41 (corresponding to glycerol at RT) to approximately 25 (corresponding to ethanol at RT), while the subcritical regime is already fairly apolar, and the supercritical regime fully apolar (Scheme 1C). Moreover, the maximal acidity and basicity of water through its autoprotolysis are found at approximately 250 °C but are lower at both lower and higher *T*. Thus, around *T*_r_≈250 °C Brønsted acid/base catalysis is provided by the medium itself, which is convenient for many reactions, including condensations. As indicated by prior work,[^23^, ^24^, ^25^, ^26^, ^27^, ^28^] and further substantiated through this contribution, the HT range of approximately 250 °C seems privileged for generating heteroaromatics through condensations, possibly enabled through an optimal combination and interplay of the relevant physicochemical characteristics of the medium.

Water is the greenest possible solvent, as it is safer for the scientist and for the environment than any other medium.^[32]^ Therefore, the different regimes of HTW have been exploited to different extent in organic synthesis. The majority of studies focused on the sub‐ and supercritical regimes (sometimes collectively called “superheated water”). These studies include hydrolyses, dehydrations, and oxidations of small organic compounds,^[33]^ and waste/biomass valorization.[^31^, ^34^] Reactions involving /heteroaromatic compounds in the super‐/subcritical regimes are limited to stability studies, showing that heteroaromatic compounds are initially stable, yet, with time undergo aquathermolysis towards open‐chain derivatives.^[35]^ Furthermore, a small number of works report the actual synthesis of heteroaromatics in the moderate HT regime (RT to ≈150 °C), including pyrazoles, pyrazolones, cromenes, phenoxythiazines, triazoles, and benzothiazoles.[^36^, ^37^] The moderate reported conditions of these syntheses seem suboptimal, for the, as we believe, privileged temperature range of approximately 250 °C for forming heteroaromatics in water. This is substantiated through all these reports employing highly polar starting compounds and only generating fairly polar products, and resulting in solely moderate to good yields.

In summary, sub‐ and supercritical water are often not amenable to heteroaromatics synthesis for degradation through aquathermolysis, and furthermore require special equipment, which limits implementation in standard organic synthesis laboratories. On the other side, synthesis in H_2_O at moderate *T*_r_ (≲100 °C), is practicable with standard laboratory equipment, but limited to polar starting compounds. In contrast, HTS is bridging the gap. HTS is synthetically straightforward: an aqueous dispersion of the stoichiometric amounts of the starting compounds (typically, no excesses required) is heated to *T*_r_>100 °C (typically 180–250 °C) in a metal autoclave placed in an oven, or in a glass vial inside a MW oven, and simply cooled down after *t*_r_.[^24^, ^25^, ^27^, ^28^] Product recuperation is also effortless: While even apolar organic substrates can dissolve and react in the HT regime, upon cooling back to RT, H_2_O becomes a highly polar medium again, and hence the organic product phase separates from H_2_O and can be collected by simple filtration (if solid) or decantation (if liquid). Typically, quantitative yields are obtained at short reaction times (<1 h), and purification is not required at all, given the absence of other reagents such as catalysts or co‐solvents. Hence, HTS bears the potential to be an “ideal” technique for rapidly providing access to a large number of derivatives. For harvesting the potential of HTS, proof of the accessibility of further than the reported functions is required, together with proof of compatibility of HTS with organic synthesis requirements (e. g., broad scope, versatility). With this work, we have set out to provide these proofs for 2,3‐diarylquinoxalines.

## Results and Discussion

To the best of our knowledge, there is only one report of quinoxaline synthesis using water as solvent, and even at room temperature.^[21]^ Satisfactory yields for the condensation of aliphatic 1,2‐diketones with substituted *o*‐phenylene diamines are reported, but only very modest yields (<30 %) are obtained for examples employing aromatic 1,2‐diketones. In particular, the reaction between *o*‐phenylendiamine (*o*‐PDA) and 4,4′‐dimethoxybenzil to form quinoxaline **1** (Table 1) is obtained with only 10 % yield. The authors attribute this to the low solubility of aromatic diketones in RT water. Considering that the polarity of liquid water decreases with *T*, that is, high‐temperature water is polarity‐wise more similar to organic solvents, we hypothesized that H_2_O at HT conditions might be a suitable medium to obtain quinoxalines where aromatic diketones are employed as starting materials.

**Table 1:** Screening of reaction conditions for synthesizing quinoxaline **1**.

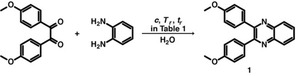					
Entry	*c* [mol L^−1^]	*T*_r_^[a]^ [°C]	*t*_r_ [min]	Additive	Yield^[b]^ [%]
1	0.02	110	10	–	4
2	0.02	150	10	–	29
3	0.02	200	10	–	56
4	0.02	230	10	–	61
5	0.01	230	5	–	38
6	0.02	230	5	–	51
7	0.04	230	5	–	65
8	0.20	230	5	–	82
9	0.50	230	5	–	83
10	1.00	230	5	–	86
11	0.20	230	10	–	88
12	0.20	230	30	–	94
13	0.20	230	60	–	*q*
14	0.02	230	10	HOAc (10 equiv.)	89
15	0.02	230	10	DIPEA	39
16	0.20	230	10	HOAc (4.3 equiv.)	*q*
17	0.20	230	10	HOAc (2.6 equiv.)	97
18	0.20	230	10	HOAc (1.0 equiv.)	97
19	0.20	230	10	oxalic acid (4.3 equiv.)	33
20	0.20	230	10	oxalic acid (2.6 equiv.)	47
21	0.20	230	10	oxalic acid (1.0 equiv.)	75
22	0.20	230	10	oxalic acid (0.1 equiv.)	86
23	0.20	230	10	oxalic acid (0.05 equiv.)	90
24	0.20	230	10	propionic acid (4.3 equiv.)	*q*
25	0.20	230	10	propionic acid (2.6 equiv.)	95
26	0.20	230	10	propionic acid (1.0 equiv.)	97

[a] *P* at 230 °C: 20–22 bar, at 200 °C: 15–20 bar, at 150 °C: 15–20 bar, and at 110 °C: max. 5 bar. [b] Determined by ^1^H NMR spectroscopy; *q*=quantitative conversion.

In order to determine the most suitable conditions for synthesizing quinoxalines hydrothermally, we selected the reaction between *o*‐PDA and 4,4′‐dimethoxybenzil to form 2,3‐bis(4‐methoxyphenyl)quinoxaline (**1**) as model reaction. Equimolar quantities of the starting compounds were dispersed in H_2_O at RT and heated to the indicated temperatures. Experiments at different *T*_r_, *t*_r_, and concentrations (*c*) of the starting materials were conducted. The tested conditions are summarized in Table 1.

In all experiments, the aqueous dispersion of the starting compounds in 1 : 1 ratio was filled in a microwave vial and heated to *T*_r_ as fast as possible (generally within 2 min). Then, the reaction mixture was kept at *T*_r_ for the specified *t*_r_ and cooled back to RT (see the Supporting Information). After cooling the reaction mixture, the crude products were isolated by filtration and analyzed directly by ^1^H NMR spectroscopy to monitor the outcome of the reaction. Note that since *o*‐PDA is soluble in H_2_O even at RT, any excess is washed away during the product isolation by filtration and hence unreacted *o*‐PDA is not found in the crude product mixture. The crude products of all experiments were mixtures exclusively composed of the starting compound 4,4′‐dimethoxybenzil and the target quinoxaline **1**, which can be easily differentiated by ^1^H NMR experiments in DMSO‐*d*_6_. Particularly, the methoxy protons in the 4,4′‐dimethoxybenzil appear as a singlet at *δ*_H_=3.87 ppm (s, 6H), whereas the same protons in quinoxaline **1** appear at *δ*_H_=3.78 ppm (s, 6H) (see the Supporting Information for spectra). First, the influence of *T*_r_ was evaluated to determine the lowest required *T* for obtaining the highest possible yield of quinoxaline **1** (Table 1, entries 1–4). The results show that the proportion of quinoxaline **1** in the crude product increases with *T*_r_. Interestingly, the proportion of **1** is only 4 % at *T*_r_=110 °C. This suggests that *T*_r_ slightly above the boiling point of water are not sufficient, but that indeed HT conditions are needed to complete the formation of **1**. The best result in our screening was obtained at *T*_r_=230 °C with 61 % of **1**. We next tested different concentrations between 0.01 and 1 mol L^−1^ at this *T*_r_ (entries 5–10). The quantity of starting materials showed a significant and counterintuitive effect on the yield of **1**, which saturated at around 80 % at 0.2 mol L^−1^. This was surprising as we would have expected smaller amounts of starting compounds to react more efficiently.

An even higher *c* (1 mol L^−1^, Table 1, entry 10) did not further improve the yield, but did also not decrease it. Then, experiments at different *t*_r_ were conducted (entries 8, 11–13), revealing that after *t*_r_=1 h the crude reaction product is composed exclusively of quinoxaline **1** (quantitative yield).

Interestingly, the ^1^H and ^13^C NMR spectra of the crude product indicate that **1** is obtained without major impurities. While pure **1** is typically of white color, the solid product showed a dark brown color, suggesting the presence of impurities at trace level. Since the reaction mixture contains only H_2_O, 4,4′‐dimethoxybenzil and *o*‐PDA, impurities would have to arise from their degradation, decomposition or side reactions. Aromatic diamines such as *o*‐PDA are indeed known to generate strongly colored products by oxidative autocondensation to oligoimine species.[^25^, ^38^] We think that such species are also the origin of the colored impurity we find here. Further purification either by reprecipitating in appropriate solvents or flash column chromatography allowed for obtaining quinoxaline **1** with 87 % yield. Based on this screening, the best conditions to obtain **1** in nothing but water are *T*_r_=230 °C, *t*_r_=1 h and *c*=0.2 mol L^−1^ with equimolar stoichiometry of starting compounds. This result is surprising, since, to our knowledge, the literature lacks procedures for synthesizing highly‐conjugated heteroaromatic scaffolds in solely water without acidic/basic promoters or further catalysts. Yet, we were curious to see if the presence of promoters could speed up the HTS of **1** even further.

On the one hand, previous work has shown that the HTS of rylene bisimides, that is, the formation of the 6‐membered imide heterocycle by cyclocondensation, is accelerated in the presence of *N,N*‐diisopropylethylamine (DIPEA).^[24]^ On the other hand, acetic acid (HOAc) is a classic solvent to synthesize quinoxalines in reflux for several hours, and has also been employed as catalyst for quinoxaline synthesis in organic solvents. Hence, we decided to test the amenability of the HTS of **1** to both Brønsted acid and base catalysts using both DIPEA and HOAc.

Interestingly, the presence of DIPEA decreased the yield of quinoxaline **1** from 61 to 39 % in the crude product, whereas the presence of acetic acid increased the yield to 89 % (Table 1, entries 14 and 15). Intrigued by the remarkable influence of acid catalyst on the reaction yield, we performed further experiments with different amounts of HOAc. To our delight, quinoxaline **1** could also be obtained at complete conversion in only *t*_r_=10 min at *T*_r_=230 °C in 5 % HOAc (4.3 equiv.) (entry 16). Lower amounts of HOAc still yield quinoxaline **1** with remarkable yields (97 %, entries 17 and 18), yet 4,4′‐dimethoxybenzyl still can be observed in the ^1^H NMR spectra of the crude products. Encouraged by these results, experiments with oxalic acid were performed (entries 19–23). First, the higher acidity of oxalic acid (p*K*_a1_=1.27 and p*K*_a2_=3.86) seemed promising and second, oxalic acid is industrially derived from biomass, which makes it a sustainable choice. Interestingly, HOAc outperforms oxalic acid at the tested concentrations (entries 16 and 19, 17 and 20, 18 and 21). We found that the presence of ≥1.0 equiv. of oxalic acid gives lower reaction yields than the HTS experiment without any acid catalyst (entry 11), whereas experiments with 0.1 and 0.05 equiv. of oxalic acid yield quinoxaline **1** with high yields (86 and 90 % respectively, entries 22 and 23). Yet, oxalic acid is employed at such high dilution in experiments with 0.1 equiv. that these experiments are in fact more comparable to pure water as reflected by the fact that their yields are identical to those of pure water at the same *T*_r_ and *t*_r_ (entry 11). To understand the role of the p*K*_a_ values of the acid catalysts, we tested propionic acid (p*K*_a_=4.8) as a promoter and found that it performs identical to HOAc at the employed amounts (entries 24–26). We hypothesize that it is not the strength of the acid used, but that acetic acid is providing an ideal pH that maximizes the reaction yield under the tested conditions. Such privileged pH‐ranges have been also observed in other reactions of nucleophilic addition of amines to carbonyl compounds.^[39]^ Note that for the earlier mentioned dehydrating ability of HT water,^[22]^ it is in principle conceivable that the employed acids would dehydrate to the corresponding ketenes (e. g., HOAc could form ethenone H_2_C=C=O). However, studies have shown that ketene formation in high‐temperature water requires significantly higher temperatures of at least 650 K (376 °C) for detectable conversion.[^40^, ^41^] Hence, we conclude that ketene formation at the here used *T* (230 °C) is highly unlikely. To sum up, two sets of conditions were determined to synthesize quinoxaline **1** hydrothermally with quantitative yields: H_2_O as solvent, *T*_r_=230 °C, *t*_r_=1 h and *c*=0.2 mol L^−1^ (subsequently referred to as “Method A”) and *T*_r_=230 °C, *t*_r_=10 min and *c*=0.2 mol L^−1^ in 5 % HOAc (“Method B”).

**Scheme 2:**
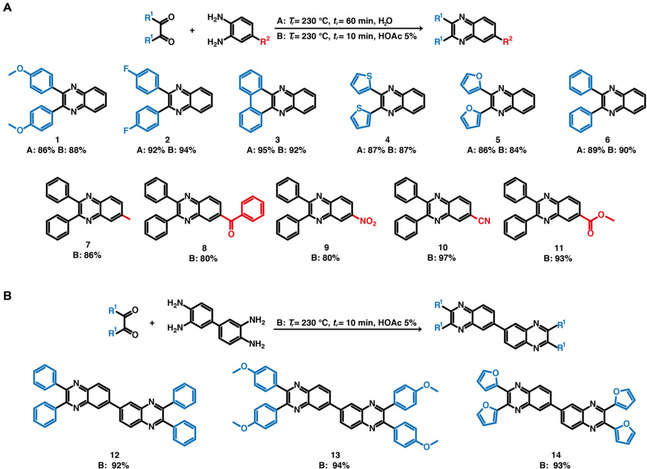
Scope of 1,2‐diketone and *o*‐PDA in HTS of (A) quinoxalines and (B) biquinoxalines (*p*_r_=20–22 bar).

To further broaden the scope of the HTS of quinoxalines, we next studied the reaction between *o*‐PDA and different 1,2‐diketones via either Method A or B, or both. Method A generated quinoxalines **2**–**6** with excellent yields ranging from 85 to 95 % (isolated yield) (see Scheme 2). In general, the crude products were all isolated by filtration and drying and, if desired, can be further purified by flash column chromatography (see the Supporting Information). All quinoxaline products were characterized by ^1^H and ^13^C NMR spectroscopy and mass spectrometry (see the Supporting Information). Confirming our previous observation that the addition of 5 % HOAc speeds up the transformation, Method B generated quinoxalines **1**–**6** in much shorter *t*_r_ without decreasing the corresponding reaction yields (Scheme 2A, Method B).

After the expansion of the scope of 1,2‐diketones, we next turned to broadening the scope of the second substrate of hydrothermal quinoxalines synthesis, using different *o*‐PDAs substituted with methyl, ketone, nitro, cyano and methyl ester functional groups in combination with benzil. We successfully obtained quinoxalines **7**–**11** in high yields using Method B (Scheme 2A). Interestingly, neither the cyano nor the methyl ester group were hydrolyzed at the employed reaction conditions. Encouraged by these results, we also tested HT conditions to synthesize biquinoxaline derivatives by reacting 3,3′‐diaminobenzidine with different diketones and could thus obtain compounds **12**–**14** with excellent yields (above 90 % in all cases, Scheme 2B). As biquinoxalines are more conjugated than their monoquinoxaline counterparts, their HTS is even more surprising.

**Scheme 3:**
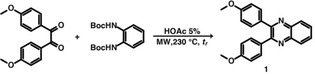
One‐pot Boc‐deprotection–quinoxaline formation sequence under HT conditions (*p*_r_=20–22 bar). Yields: 73 % with *t*_r_=10 min and 76 % with *t*_r_=30 min.

The synthesis of target molecules of higher structural complexity requires further protection and deprotection steps. While necessary, these additional reaction steps reduce the overall environmental friendliness of any synthetic route. For instance, diamines can be protected employing di‐*tert*‐butyldicarbonate (Boc_2_O) and are often even commercially available in the Boc‐protected form. The deprotection step is usually performed under strongly acidic conditions such as mixtures of TFA/CH_2_Cl_2_ or HCl in 1,4‐dioxane.^[42]^ Deprotection by heating in H_2_O at 150 °C for several hours is also possible, but rarely used.^[43]^ Since these conditions correspond to a HT treatment, we sought to perform a fully hydrothermal Boc‐deprotection‐quinoxaline formation sequence in one step, hence eliminating the necessity for intermediate isolation. To achieve this, the reaction between the Boc‐diprotected *o*‐PDA and 4,4′‐dimethoxybenzil was performed according to Scheme 3.

We found that quinoxaline **1** could be obtained with a 73 % yield (determined by ^1^H NMR spectroscopy) employing Method B (*T*_r_=230 °C, *t*_r_=10 min and *c*=0.2 mol L^−1^). Extending *t*_r_ to 30 min did not increase the yield. These results clearly show that HT conditions allow for the deprotection and quinoxaline formation at good yields in a single reaction step.

Prompted by our observation that HT synthesis of quinoxalines is catalyzed by the presence of HOAc, we tested if the inherent acidity of amine hydrochlorides as precursors would be sufficient to promote the reaction. We hypothesized that dihydrochloride derivatives would be a dual‐role starting material in the HT synthesis of quinoxalines as they could act both as source of protons to catalyze the reaction and also as source of *o*‐PDA.

**Scheme 4:**
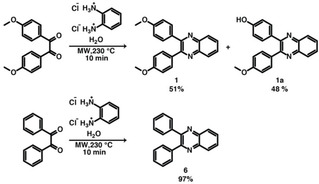
Reaction between *o*‐PDA dihydrochloride and 1,2‐diketones under HT conditions (*p*_r_=20–22 bar).

To test this hypothesis, we used the commercially available *o*‐PDA dihydrochloride (*o*‐PDA⋅2HCl) as starting material. Note that *o*‐PDA⋅2HCl has a higher solubility in water than free *o*‐PDA. Then, 4,4′‐dimethoxybenzyl and *o*‐PDA hydrochloride were reacted under HT conditions at *T*_r_=230 °C and *t*_r_ *=*10 min in nothing but water (Method A) and with 5 % HOAc (Method B), as shown in Scheme 4. Interestingly, the ^1^H NMR spectrum of the crude product formed by Method A with *t*_r_=10 min showed signals corresponding to quinoxaline **1** together with additional signals that suggested the presence of another compound in the mixture (see the Supporting Information, Figure S29). Purification of the crude reaction mixture by column chromatography allowed us to obtain quinoxaline **1** and compound **1** **a** with 51 and 48 % yield, respectively. Through ^1^H and ^13^C NMR experiments in combination with MS data, compound **1** **a** could be identified as 4‐(3‐(4‐methoxyphenyl)quinoxaline‐2‐yl)phenol. The formation of compound **1** **a** is explained by the hydrolysis of one methoxy group in quinoxaline **1**. We tested longer reaction times (*t*_r_=20 or 30 min) to deliberately hydrolyze both ether groups, yet the second methoxy group was not hydrolyzed under the tested conditions.

The crude product of the same reaction in 5 % HOAc (Method B) contained again both quinoxalines **1** and **1** **a**, and additionally the starting material 4,4‐dimethoxybenzil (see the Supporting Information, Figure S28). These findings clearly show that *o*‐PDA dihydrochloride can be employed as starting material for synthesizing quinoxalines hydrothermally in shorter *t*_r_ without the need of HOAc. Moreover, too much acid promotes the backreaction (hence remaining starting compound 4,4’‐dimethoxybenzil found by Method B). Additionally, care should be taken when acid‐labile functional groups are present in the 1,2‐diketone. We furthermore tested the use of *o*‐PDA⋅2HCl with benzil as 1,2‐diketone without acid‐labile groups towards quinoxaline **6**. Indeed, as methoxy groups are absent in benzil, ether hydrolysis cannot take place, and within *t*_r_=10 min quinoxaline **6** was obtained as only reaction product with 97 % yield. Experiments to synthesize quinoxaline **6** at lower *T*_r_ revealed that it can be obtained without considerably losing reaction yield at 200 °C, whereas 180 °C are not enough to complete the reaction in *t*_r_=10 min (see Figure S32). In summary, the amenability of *o*‐PDA⋅2HCl to the direct HTS of quinoxalines is of particular practical relevance, as (i) amine hydrochlorides are commercially available, (ii) amine hydrochlorides are much less prone to oxidation than free amines and hence have higher storability and (iii) they also provide higher water solubility than free amines.

Our versatile hydrothermal synthesis route towards a broad range of quinoxalines is also particularly environmentally friendly: We use solely water as solvent, or respectively an aqueous 5 % acetic acid solution corresponding virtually to vinegar. Our route generates the products in high to excellent yields at short reaction times, and does not rely on complex/expensive reactants or catalysts or excesses of either of the starting compounds. Our approach can also be adopted by any organic synthesis laboratory without implementing a more complex experimental set‐up. Any novel approach towards the synthesis of a known scaffold is expected to show similar or better performance compared to the reported methods in literature. In our case, this sort of comparison is a demanding endeavor as there are hundreds of synthetic procedures reported for quinoxalines. To systematically assess the performance of our hydrothermal synthesis of quinoxalines, we performed a computational analysis comparing our synthesis to all previous approaches reported in the literature.

**Figure 1:**
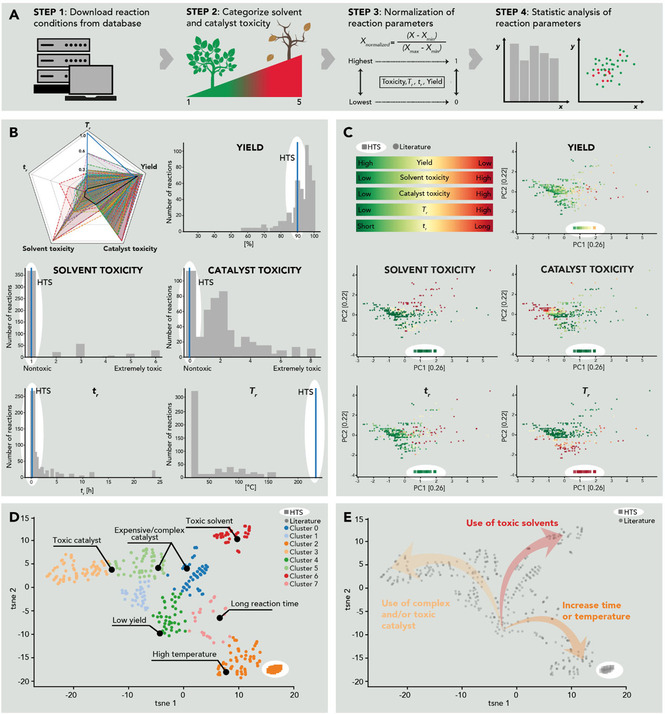
(A) Overview of the reaction parameter extraction pipeline. (B) Differences between HTS of quinoxalines and the reported literature synthesis. The spider plot depicts with dashed borders *n*=323 reactions that report all of the five parameters (solvent, catalyst, *T*_r_, *t*_r_ and yield) used in the analysis. The black solid line indicates the average of all the 323 considered reactions whereas the blue solid line represents the average of HT conditions. Spider plots for individual compounds are included in the Supporting Information (Figure S36) The different histograms were plotted considering the number of reactions that report the corresponding parameter: solvent (*n*=452), catalyst (*n*=484), *T*_r_ (*n*=479), *t*_r_ (*n*=538), yield (*n*=513). (C) Reaction space of 2,3‐diarylquinoxalines with overlay of the 5 investigated features. Green depicts a more favourable reaction parameter whereas red indicates less favourable conditions. (D) t‐SNE dimension reduction depicting clusters of favourable reaction conditions. (E) Possible reaction strategies to improve reaction yields.

To this end, we extracted all the reported syntheses for quinoxalines **1**–**14** from the commercial database Reaxys® (see Figure 1A for the data extraction and curation pipeline). A total of 1033 syntheses towards compounds **1**–**14** are reported (see the Supporting Information). As expected, the Hinsberg cyclization is the most used approach among the reported syntheses (581 reactions, 56 %) and the analysis per individual compound also shows that the Hinsberg cyclization has been the most frequent syntheses for all of the compounds except compound **4**. Only these reactions were employed in our computational analysis. The remaining syntheses comprehend a variety of reactions between *o*‐PDA analogues and variants of 1,2‐diketones (e. g., benzoins, vicinal diols, α‐substituted acetophenones). Note that our analysis aims explicitly at evaluating the reaction conditions for transforming starting compounds to products, but does not account for their toxicity. All the alternative synthesis would involve different requirements, leading to inequitable comparisons. Therefore, these alternative syntheses were not included in the analysis. To the best of our knowledge, the analysis here presented is covering the highest number of reported synthesis for the scope of compounds presented in a work.

Among the 581 reported reactions, the numbers of reported syntheses per compound are ranging from 1 to 164 (Supporting Information, Table S1). This number is relatively small with respect to the several thousand quinoxalines known to the literature. For our subsequent analysis, we included the following five, typically well‐reported reaction parameters: (i) temperature, (ii) time, (iii) yield, (iv) reaction solvent and (v) catalyst. All other parameters, such as pressure or pH, were discarded due to being overall poorly reported. We removed all incompletely annotated reactions with missing information for parameters (i)–(iii), resulting in 323 final syntheses. We assessed the toxicity of solvents and catalysts according to Byrne et al.^[32]^ and the globally harmonized system (GHS), respectively (see the Supporting Information, Figure S35 and method 5.1 for more details). Our analysis shows that HTS of quinoxalines clearly differs from classical reactions in several ways (Figure 1B).

First, the HT route uses by far the highest reaction temperature. Second, HTS belongs to the fastest reaction types. Third, our approach uses the cleanest solvent. The reported synthesis of **1**–**14** use recommended solvents such as ethanol (37 %), water (25 %), methanol (12 %), glycerol (3 %), mixtures of the above (13 %) or neat conditions (11 %). Note that several of the reported quinoxaline syntheses with alcohols as solvents require HOAc as catalyst. In contrast, our HTS approach generates high yields also in the absence of acid, provided through the intrinsic acidity of H_2_O(l) at *T*_r_≈250 °C, and can additionally be sped up through HOAc. Moreover, whereas solvents such as MeOH or EtOH seem closely related to H_2_O, they are in fact quite different. In terms of hazardousness, MeOH bears substantial flammability and toxicity risks. As media, MeOH and EtOH are significantly less polar than H_2_O: for instance, the static dielectric constant (*ϵ_r_*) of MeOH at RT is approximately 33, a value that is only matched by water at 220 °C. Therefore, obtaining quinoxalines **1**–**14** in alcohols, which are solvents of medium polarity, is much less surprising than synthesizing them in H_2_O. Moreover, our combination of solvent/catalyst is one of the cleanest ones and among the considered reactions, only the water/PEG600 combination is comparably green. The yield of the HTS is overall comparable with other reported reactions (see the Supporting Information, Figure S36 for individual compound results). Note however, that the literature is expected to be strongly biased towards positive results in this regard.

The distinct characteristics of HTS compared to classical synthesis routes can be further assessed using a principal component analysis (PCA), which maps the five dimensions of each reaction to a two‐dimensional point, such that the overall variance across all reactions is maximized. Figure 1C shows the resulting PCA mapping with reactions from the literature indicated as circles and HTS as squares. Each panel shows the distribution of one particular reaction parameter across all reactions, highlighting generally favorable conditions in green and unfavorable ones in red. The separation of the HTS cluster from all other reactions exemplifies the distinct HT reaction conditions. This separation is mostly driven by the high *T*_r_ used in HTS and would likely be even larger if reaction pressure would also be considered. We can further extract interesting relationships between the reaction parameters by comparing different panels. We observe, for example, that the yield seems to correlate with the toxicity of the used catalyst, as indicated by the reversed red and green colored areas in the yield and catalyst panels of Figure 1C, whereas longer reaction times alone do not appear to systematically relate to higher yields. The correlation between time and temperature (and to a lesser extent solvent toxicity) might indicate that increasing both simultaneously occurs more frequently in reported reactions.

Next, we investigated similarities between different reactions using the dimension reduction technique t‐SNE^[44]^ (T‐distributed stochastic neighborhood embedding) and subsequently applying a *k*‐means clustering (*k*=8) to identify clusters of reactions with similar reactions conditions (see Figure 1D, clusters of similar reactions are indicated by the same color). Interestingly, we found that the reaction conditions for the same chemical compound are generally not similar for different synthesis routes (Supporting Information, Figure S38). This may reflect a tendency in the published literature to optimize the reaction conditions only for one specific compound and subsequently apply these conditions to a series of additional compounds. A closer examination of the different clusters of reactions with similar conditions (see the Supporting Information, Figure S39 for individual cluster results and feature overlay) reveals that reactions with optimal yield can be typically assigned to the usage of (i) higher *T*_r_ (cluster 0), (ii) increasing *t*_r_ (cluster 7), (iii) usage of toxic solvents (cluster 4) or catalyst (cluster 3 and 0) or (iv) addition of complex catalyst to the reaction (cluster 2 and 5). Cluster 6 corresponds to reaction conditions that do not apply any of the previously mentioned methods, typically resulting in poor yield. From a data mining perspective, such “negative data” are extremely valuable, as they allow for extracting trends for systematically improving reaction conditions (Figure 1E). Our analysis indicates that the synthesis of quinoxalines at RT and without toxic catalysts and/or solvents works poorly. Higher yields can be obtained by using (i) toxic solvents, (ii) toxic/complex catalysts or (iii) increasing the temperature often in combination with time. Intriguingly, HTS follow all three approaches except for the toxicity aspect: (i) HT conditions are intrinsically found at high temperatures, (ii) high‐temperature water behaves like an organic solvent (in terms of polarity and viscosity), however without being toxic, and (iii) HT water can act as both Brønsted acid and base through its increased ionic product.

Finally, we explored a potential application of our newly synthesized compounds. Quinoxalines are receiving a growing interest due to their fluorescence properties. In solution, quinoxalines have been shown to display solvatofluorochromic behavior, and some representatives also exhibit aggregation‐induced emission in the solid state.[^7^, ^8^] Therefore, we investigated the fluorescence properties of all the synthesized quinoxalines **1**–**14**. The results show that monoquinoxalines **1**, **4**, **5**, **7**, and **11** exhibit blue fluorescence and the intensity is higher in compounds with electron donating groups such as methoxyphenyl, furane, and thiophene rings at positions C‐2 and C‐3 of the quinoxaline ring (Supporting Information, Figure S40). The remaining monoquinoxalines did not show fluorescence even at millimolar concentration. Moreover, all biquinoxalines (**12**–**14**) exhibited blue fluorescence.

**Figure 2:**
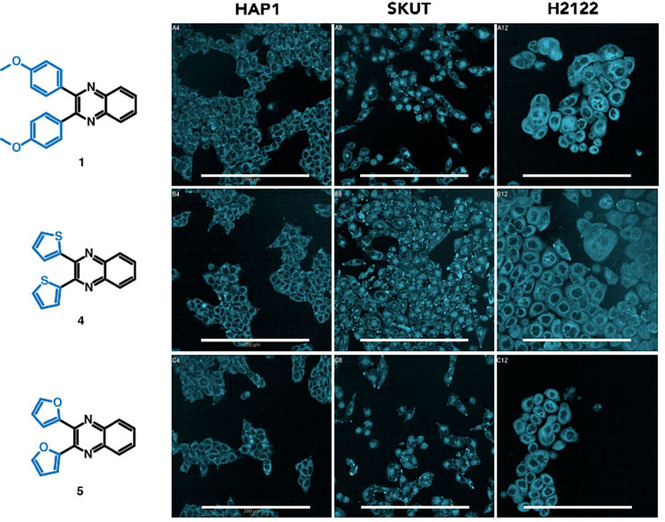
Fluorescence microscopy images of the indicated cell lines after treatment with solutions 1.5 μm in DMSO of quinoxalines **1**, **4** and **5** (scale bar 200 μm). Results for all of the compounds are included in the Supporting Information (Figure S41).

Given that quinoxalines are well‐known to depict biological activity, we decided to investigate compounds **1**–**12** as cell stains. Fluorescent probes based on small compounds are extremely useful in fluorescence microscopy as they allow imaging biochemical processes with remarkable sensitivity and temporal resolution.^[45]^ Important properties of fluorescent probes for cell staining are cell permeability, high brightness and photostability. The cell staining capacity of compounds **1**–**12** was tested at different concentrations against a panel of three different cell lines: HAP1 (near‐haploid human cells), SKUT1 (human uterine leiomyosarcoma cells) and H2122 (human lung cancer cells). Confocal microscopy images show that among the tested quinoxalines **1**, **4**, **5** are cell permeable and sufficiently bright at a micromolar concentration (Figure 2). They exhibit blue emission and appear nicely distributed throughout the cytoplasm in all three studied cell lines. While some properties of the tested compounds such as solvatochromism are low compared to previously reported quinoxalines,[^46^, ^47^] we believe that the cell permeability and fluorescence of these relatively simple scaffolds is already promising. Further work focusing on derivatization of these scaffolds towards higher fluorescence intensities and longer wavelengths of emission is currently ongoing.

## Conclusion

With this work, we report the synthesis of quinoxalines from 1,2‐diketones and *o*‐phenylendiamine derivatives in nothing but high‐temperature water. The very short reaction times (30 min) make our approach highly practical. Catalysts are not necessary, but if desired, the quinoxaline condensation can be accelerated by 5 % HOAc to reaction times of ≤10 min. The target quinoxalines are obtained in high yields and we showed that the reaction is tolerant to a broad array of functional groups. Furthermore, *o*‐phenylendiamine (*o*‐PDA) dihydrochloride derivatives are suitable substrates that do not only provide better solubility, but also generate an acidic environment in situ, avoiding the addition of an acid catalyst whilst autogenously speeding up the reaction. In addition, a sequence of Boc‐deprotection of amines could be coupled with the formation of the quinoxaline ring in a one‐pot fashion in nothing but hot water. To date, hydrothermal (HT) synthesis of highly conjugated organic compounds has been limited to amides,^[23]^ cyclic imides,^[24]^ perinones,^[28]^ and benzimidazoles.^[26]^ With this work, we extend the scope of HT synthesis to another heteroaromatic moiety. Our large‐scale computational analysis of what we believe are to date all synthetic means towards quinoxalines **1**–**14** revealed that the hydrothermal approach is particularly green. Nevertheless, in an effort to make hydrothermal synthesis (HTS) even greener, future work will focus on exploring HTS in flow, which is expected to lower reaction times even further, and to employ starting materials with lower toxicity. Finally, we show that the synthesized quinoxalines are promising candidates for developing cell staining probes. The wavelengths of emission are mainly blue and their intensities are low compared to reported dyes. Yet, the cell permeability and fluorescence inside cells observed were promising in the performed cell staining assays. In the future, we will further explore these scaffolds for cell staining.

## Experimental Section

### Materials and general methods

All chemicals were obtained from TCI or Sigma Aldrich and used without further purification. Distilled water was employed in all the experiments. The reactions were conducted in an Anton Paar Monowave 400 microwave reactor, employing G30 and G10 glass vials. ^1^H and ^13^C NMR spectra were recorded on a Bruker Avance DRX‐400 spectrometer. Samples were dissolved in CDCl_3_ (*δ*_H_=7.26 ppm, *δ*_C_=77.0 ppm) or DMSO‐*d*_6_ (*δ*_H_=2.50 ppm) and the spectra for all compounds are included in the Supporting Information.

### Hydrothermal synthesis of quinoxalines

**General procedure for Method A**: In a glass vial with a magnetic stirrer, the respective 1,2‐diketone (0.4 mmol) and the corresponding *o*‐phenylendiamine (0.4 mmol) were suspended in water (2 mL). Then, the glass vial was placed into the cavity of the microwave reactor and the suspension was heated up as fast as possible to 230 °C. The maximum pressures observed ranged between 20–22 bar. The temperature was maintained for 60 min and afterwards the mixture was cooled down to RT. The crude product was filtered and dried in an oven. If necessary, the compounds could be further purified by dissolving in acetone or THF and pouring the mixture into water, or by flash column chromatography (SiO_2_), eluting with petroleum ether/ethyl acetate 6 : 4.

**General procedure for Method B**: In a glass vial with a magnetic stirrer, the respective 1,2‐diketone (0.4 mmol) and the corresponding *o*‐phenylendiamine (0.4 mmol) were suspended in a solution of 5 % HOAc (2 mL). Then, the glass vial was placed into the cavity of the microwave reactor and the suspension was heated up as fast as possible to 230 °C. After 10 min, the mixture was cooled down, filtered and dried. The crude samples could be purified as described for Method A.

### Database search and computational analysis

The complete computational analysis was performed using Phyton v3.7.2. The employed code can be accessed as Jupyter notebooks via Github under the following url: UnterlassLab github page https://github.com/UnterlassLab/Computational_Analysis_HTS_2‐3‐diarylquinoxalines. A step‐by‐step description of the analysis is included in the Supporting Information (Data acquisition and feature normalization, individual spider plots for each compound, dimensionality reduction techniques and *k*‐means cluster analysis).

### Fluorescence microscopy experiments

The cell lines HAP1 (near‐haploid human cells), SKUT (human uterine leiomyosarcoma cells) and H2122 (human lung cancer cells) were grown in 96 well plates (15000 cells per well). The cells were maintained under 5 % CO_2_ atmosphere at 37 °C. For cell staining experiments, 100 mm stock solutions of compounds in DMSO were diluted to 10 mm by hand and cells were treated with diluted solutions of the compounds to get final concentrations of 45, 15, 5 and 1.5 μm. Cells were incubated for 45 min at 37 °C under CO_2_ atmosphere and live‐imaged using an Opera Phenix high‐content fluorescence microscope (PerkinElmer) with 40X objective. Fluorescence microscopy images for all the compounds are included in the Supporting Information.

## Conflict of interest

The authors declare no conflict of interest.

## Supporting information

As a service to our authors and readers, this journal provides supporting information supplied by the authors. Such materials are peer reviewed and may be re‐organized for online delivery, but are not copy‐edited or typeset. Technical support issues arising from supporting information (other than missing files) should be addressed to the authors.

Supplementary
