# Supplementary material for: Green Hydrothermal Synthesis of Fluorescent 2,3‐Diarylquinoxalines and Large‐Scale Computational Comparison to Existing Alternatives
Source: ChemSusChem. 2021 Mar 26;14(8):1853–63. doi: 10.1002/cssc.202100433 (PMC8252754; doi:10.1002/cssc.202100433)
Supplement: Supplementary file 1 — Supplementary [file CSSC-14-1853-s001.pdf]

ChemSusChem

Supporting Information

Green Hydrothermal Synthesis of Fluorescent 2,3-Diarylquinoxalines and Large-Scale Computational Comparison to Existing Alternatives

Fabián Amaya-García, Michael Caldera, Anna Koren, Stefan Kubicek, Jörg Menche, and Miriam M. Unterlass*© 2021 The Authors. ChemSusChem published by Wiley-VCH GmbH. This is an open access article under the terms of the Creative Commons Attribution License, which permits use, distribution and reproduction in any medium, provided the original work is properly cited.

Table of Contents

1. **General Procedures**……………………………………………………………………………………………………..2

2. **Screening of conditions**…………………………………………………………………………………………………..2
   2.1. Model reaction…………………………………………………………………………………………………...2
   2.2. Experiments at different temperatures………………………………………………………………………..2
   2.3. Experiments at different concentrations……………………………………………………………………...3
   2.4. Experiments with different reaction times……………………………………………………………………...4
   2.5. Experiments with different amounts of carboxylic acids…………………………………………………….4
   2.6. NMR spectra of crude quinoxaline (1)…………………………………………………………………………..5

3. **Synthesis and characterization of compounds**…………………………………………………………………..6

4. **Overview of number of reported quinoxalines in literature and solvents employed in their synthesis**…….25

5. **Computational analysis of the reaction space**……………………………………………………………………26
   5.1. Data acquisition and feature normalization…………………………………………………………………..25
   5.2. Individual spider plots for each compound……………………………………………………………………31
   5.3. Dimensionality reduction techniques……………………………………………………………………………32
   5.4. K-means cluster analysis………………………………………………………………………………………….33

6. **Fluorescence measurements and fluorescence microscopy experiments**……………………………………35
   6.1. Fluorescence measurements of quinoxalines **1-14**………………………………………………………….35
   6.2. Fluorescence microscopy experiments…………………………………………………………………………..35

1. General procedures

All chemicals were obtained from TCI or Sigma Aldrich and used without further purification. Distillated water was employed in all the experiments. All the assisted microwave reactions were conducted in a reactor Anton Paar Monowave 400, employing G30 and G10 glass vials. Column chromatography purifications were conducted with silica gel as stationary phase. ^{1}H and ^{13}C NMR spectra were recorded on a Bruker Avance DRX-400 spectrometer. Samples were dissolved in $CDCl_3$ (δ_H = 7.26 ppm, δ_C = 77.0 ppm) or DMSO-d$_6$ (δ_H = 2.50) and the chemical shifts are reported with the solvent residual signal as reference. The coupling constants are reported in Hertz. Mass spectrometry experiments were carried out in Spectrometer. UV/VIS absorption spectra and fluorescence emission spectra were recorded in a Perkin Elmer Lambda 750 spectrometer and an Edinburgh FLS920, respectively.

2. Screening of conditions

2.1. Model reaction

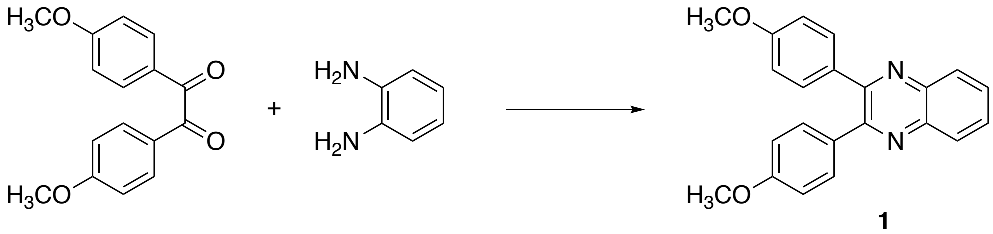

1

4,4´-dimethoxybenzyl: ^{1}H NMR (400 MHz, DMSO-*d*$_6$) δ_H 7.86 (d, *J* = 8.9 Hz, 4H)*, 7.13 (d, *J* = 8.8 Hz, 4H), 3.87 (s, 6H).

Quinoxaline (**1**): ^{1}H NMR (400 MHz, DMSO-d$_6$): δ_H 8.09 (dd, *J* = 6.4, 3.4 Hz, 2H), 7.82 (dd, *J* = 6.4, 3.4 Hz, 2H)*, 7.45 (d, *J* = 8.8 Hz, 4H), 6.93 (d, *J* = 8.8 Hz, 4H), 3.78 (s, 6H); ^{13}C NMR (100 MHz, DMSO-d$_6$) δ_C 159.8, 152.6, 140.3, 131.3, 131.1, 129.9, 128.6, 113.6, 55.2.
*Overlapped signals

2.2. Experiments at different temperature

<u>t_r = 10 min, C_M = 0.02 mol/L</u>

^{1}H NMR (400 MHz, DMSO-d$_6$)

110 °C 150 °C 200 °C 230 °C

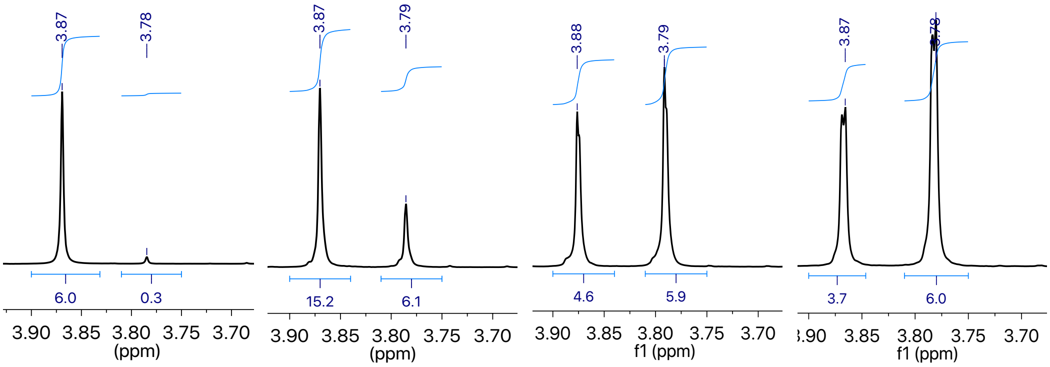

T_r (°C)	% **1**
110	4
150	29
200	56
230	61

2.3. *Experiments at different concentrations*

T_r = 230 °C, t_r = 5 min

^{1}H NMR (400 MHz, DMSO-d_6)

0.01 mol/L

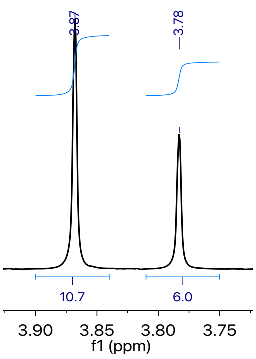

0.02 mol/L

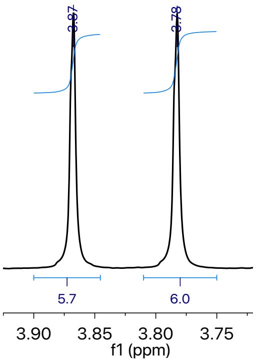

0.04 mol/L

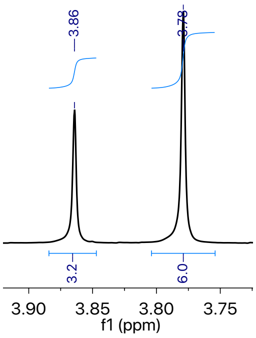

0.2 mol/L

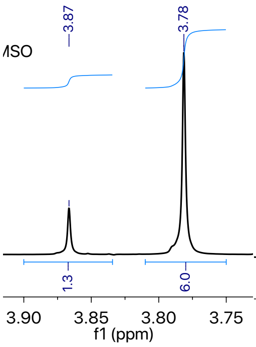

0.4 mol/L

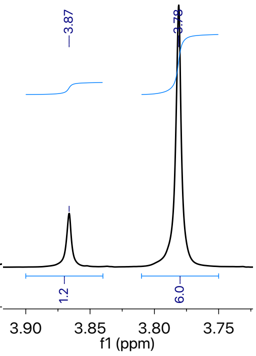

1.0 mol/L

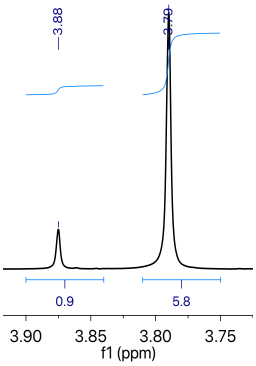

C_M (mol/L)	% **1**
1.00	87
0.50	83
0.20	82
0.04	65
0.02	51
0.01	38

2.4. *Experiments with different reaction times*

T_r = 230 °C, C_M = 0.2 mol/L

^{1}H NMR (400 MHz, DMSO-d_6)

5 min

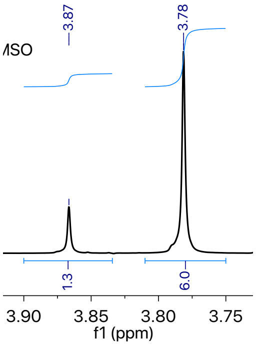

10 min

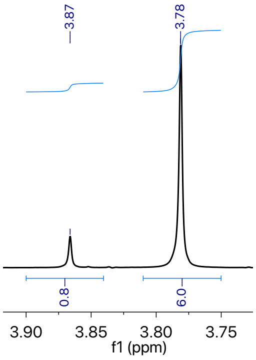

30 min

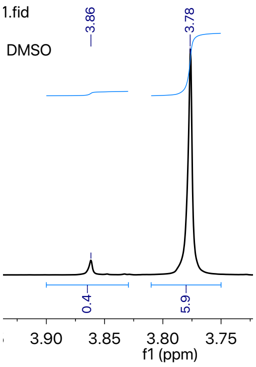

60 min

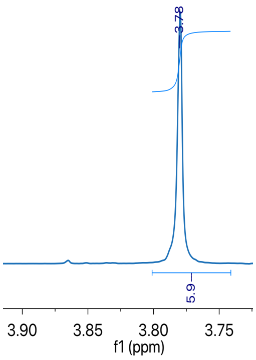

t_r (min)	% **1**
5	82
10	88
30	94
60	Complete

2.5. Experiments with different amounts of carboxylic acids

Oxalic acid : T_r = 230 °C, C_M = 0.2 mol/L
^{1}H NMR (400 MHz, DMSO-d_6)

4.6 equiv. | 2.3 equiv. | 1.0 equiv. | 0.5 equiv. | 0.1 equiv.

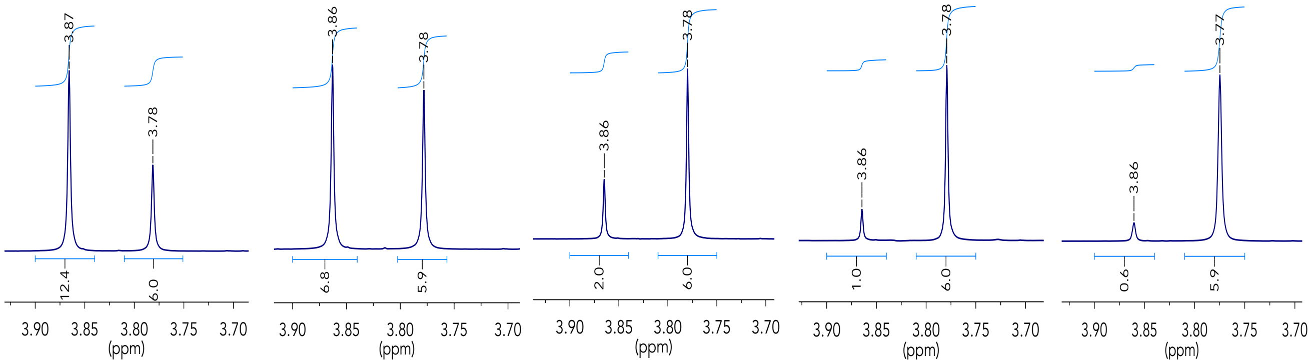

Equiv. Oxalic acid	% **1**
4.6	33
2.3	47
1.0	75
0.5	86
0.1	90

Propionic acid: T_r = 230 °C, C_M = 0.2 mol/L
^{1}H NMR (400 MHz, DMSO-d_6)

4.6 equiv. | 2.3 equiv. | 1.0 equiv.

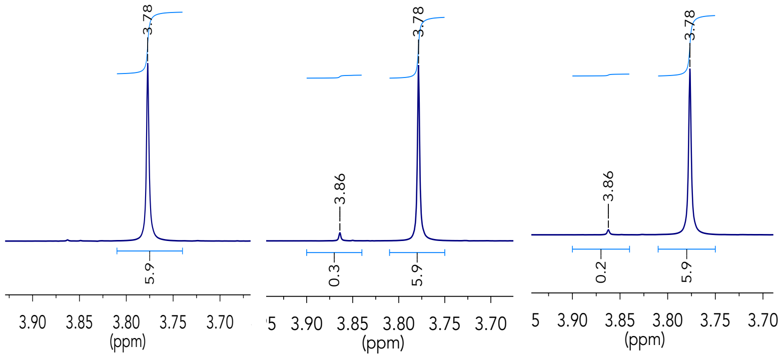

Equiv. Propionic acid	% **1**
4.6	q
2.3	95
1.0	97

Acetic acid : T_r = 230 °C, C_M = 0.2 mol/L
^{1}H NMR (400 MHz, DMSO-d_6)

4.6 equiv. | 2.3 equiv. | 1.0 equiv.

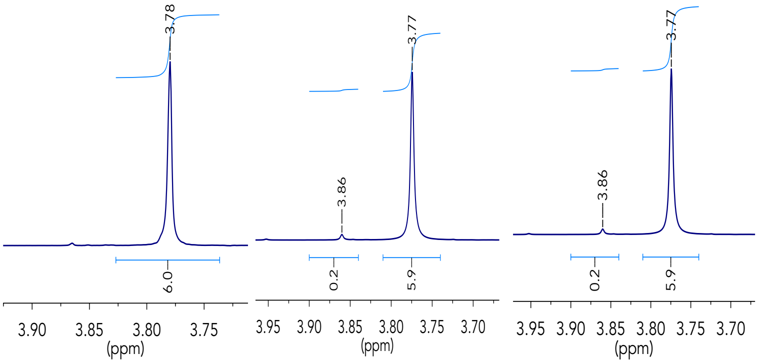

Equiv. Acetic acid	% **1**
4.6	q
2.3	97
1.0	97

2.6. ^{1}H and ^{13}C NMR of crude Quinoxaline (**1**)

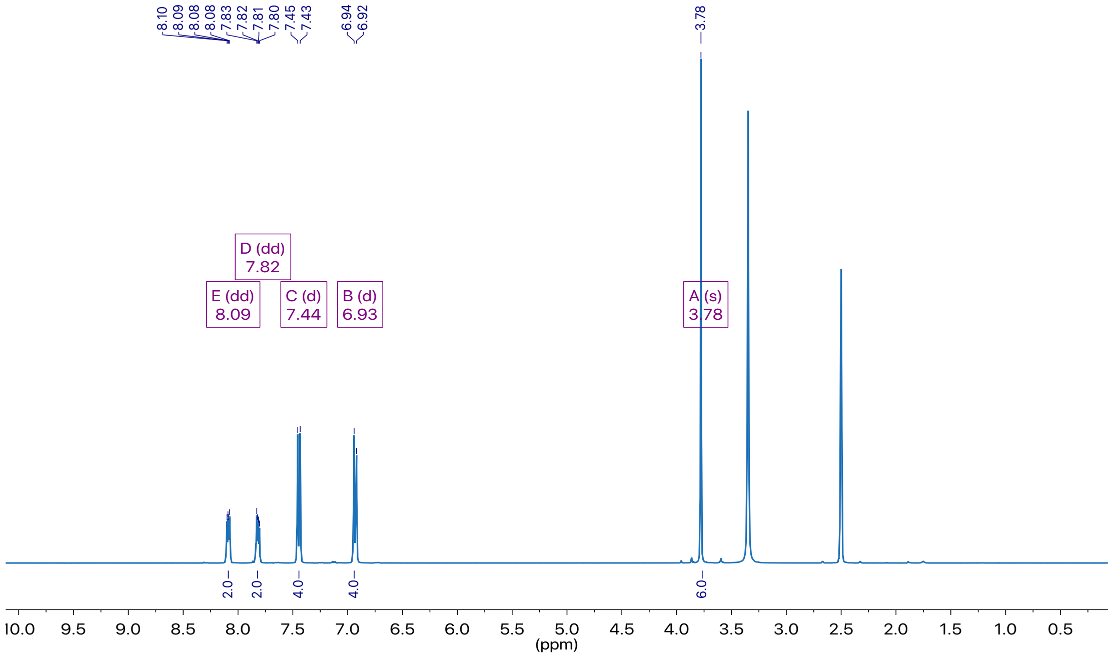

Figure SI1. ^{1}H NMR of the crude quinoxaline **1**

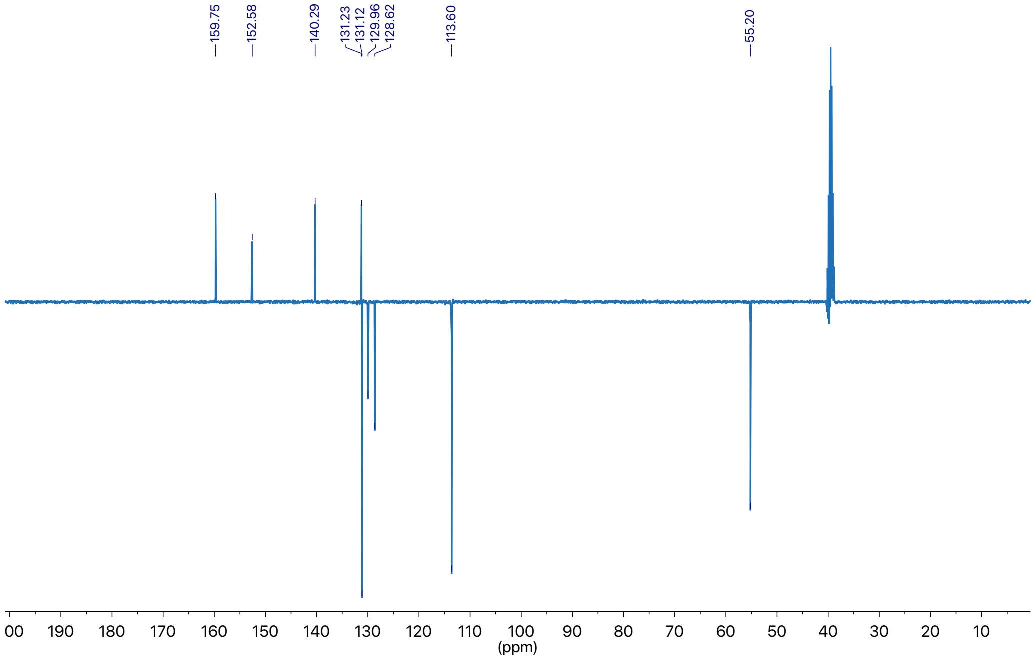

Figure S2. APT of the crude quinoxaline **1**

3. Synthesis and characterization of compounds

General procedure for the synthesis of quinoxalines in water: Method A

In a glass vial with a magnetic stirrer, the corresponding 1,2-diketone (0.4 mmol) and *o*-phenylendiamine (0.4 mmol) are suspended in water (2 mL). Then, the glass vial is placed into the cavity of the microwave reactor and the suspension is heated as fast as possible to 230°C. Maximum pressure observed ranged between 20-22 bar. Temperature is maintained for 60 minutes and afterwards the mixture is cooled down to room temperature. The crude product is filtered and dried in an oven. If necessary, the compounds can be further purified by dissolving in acetone or THF and pouring the mixture into water or flash column chromatography (SiO_2) eluting with petroleum ether:EtOAc 6:4.

General procedure for the synthesis of quinoxalines in solution of 5% AcOH: Method B

In a glass vial with a magnetic stirrer, the corresponding 1,2-diketone (0.4 mmol) and *o*-phenylendiamine (0.4 mmol) are suspended in a solution of 5% acetic acid in water (2 mL). Then, the glass vial is placed into the cavity of the microwave reactor and the suspension is heated as fast as possible to 230°C. After 10 minutes, the mixture is cooled down, filtered and dried. The crude samples can be purified as described for the experiments in water.

2,3-bis(4-methoxyphenyl)quinoxaline (1)

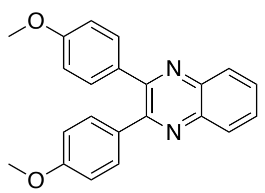

Yellow solid; Method A: 86% Method B: 88% yield; ^{1}H NMR (400 MHz, $CDCl_3$) δ_H 8.13 (dd, *J* = 6.4, 3.4 Hz, 2H), 7.72 (dd, *J* = 6.4, 3.4 Hz, 2H), 7.50 (d, *J* = 8.8 Hz, 4H), 6.88 (d, *J* = 8.8 Hz, 4H); ^{13}C NMR (100 MHz, $CDCl_3$) δ_C 160.2, 153.2, 141.2, 131.7, 131.3, 129.6, 129.0, 113.8, 55.3.

2,3-bis(4-fluorophenyl)quinoxaline (2)

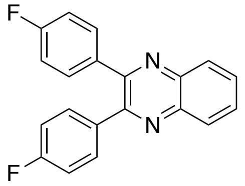

White solid; Method A: 92% Method B: 94% yield; ^{1}H NMR (400 MHz, $CDCl_3$) δ_H 8.16 (dd, *J* = 6.4, 3.4 Hz, 2H), 7.79 (dd, *J* = 6.4, 3.4 Hz, 2H), 7.51 (m, 4H), 7.06 (t, *J* = 8.7 Hz, 4H); ^{13}C NMR (100 MHz, $CDCl_3$) δ_C 164.6, 162.1, 152.3, 141.3, 135.2, 135.1, 132.0, 131.9, 130.4, 129.3, 115.8, 115.6.

dibenzo[a,c]phenacine (3)

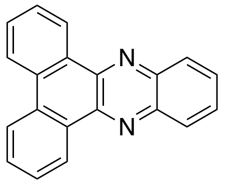

Yellow solid; Method A: 95% Method B: 92% yield; ^{1}H NMR (400 MHz, $CDCl_3$) δ_H 9.38 (dd, *J* = 7.8, 1.6 Hz, 2H), 8.53 (d, *J* = 7.8 Hz, 2H), 8.31 (dd, *J* = 6.5, 3.4 Hz, 2H), 7.84 (dd, *J* = 6.5, 3.4 Hz, 2H), 7.75 (dtd, *J* = 19.4, 7.2, 1.4 Hz, 4H); ^{13}C NMR (100 MHz, $CDCl_3$) δ_C 142.6, 142.3, 132.2, 130.4, 129.9, 129.6, 128.1, 126.4, 123.0.

2,3-bis(thiophen-2-yl)quinoxaline (4)

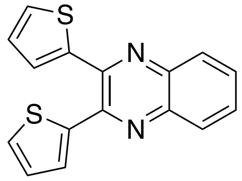

Yellow solid; Method A: 87% Method B: 87% yield; ^{1}H NMR (400 MHz, $CDCl_3$) δ_H 8.08 (dd, *J* = 6.4, 3.4 Hz, 1H), 7.72 (dd, *J* = 6.4, 3.4 Hz, 1H), 7.50 (dd, *J* = 5.1, 1.1 Hz, 1H), 7.25 (dd, *J* = 3.7, 1.1 Hz, 1H), 7.04 (dd, *J* = 5.1, 3.7 Hz, 1H); ^{13}C NMR (100 MHz, $CDCl_3$) δ 146.82, 141.62, 140.80, 130.28, 129.48, 129.03, 127.72.

2,3-bis(furan-2-yl)quinoxaline (5)

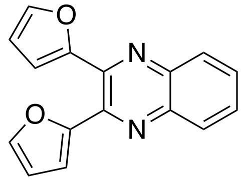

Brown solid; Method A: 86% Method B: 84% yield; ^{1}H NMR (400 MHz, $CDCl_3$) δ_H 8.14 (dd, *J* = 6.4, 3.4 Hz, 2H), 7.75 (dd, *J* = 6.4, 3.4 Hz, 1H), 7.63 (dd, *J* = 1.8, 0.7 Hz, 1H), 6.66 (dd, *J* = 3.5, 0.7 Hz, 1H), 6.57 (dd, *J* = 3.5, 1.8 Hz, 1H); ^{13}C NMR (100 MHz, $CDCl_3$) δ_c 150.9, 144.4, 142.8, 140.8, 130.6, 129.3, 113.2, 112.1.

2,3-diphenylquinoxaline (6)

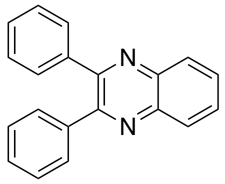

White solid; Method A: 89% Method B: 90% yield; ^{1}H NMR (400 MHz, $CDCl_3$) δ 8.19 (dd, *J* = 6.4, 3.4 Hz, 1H), 7.78 (dd, *J* = 6.4, 3.4 Hz, 1H), 7.53 (dd, *J* = 7.8, 1.8 Hz, 2H), 7.40 – 7.31 (m, 3H); ^{13}C NMR (100 MHz, $CDCl_3$) δ_c 153.6, 141.4, 139.2, 130.1, 130.0, 129.4, 129.0, 128.4.

6-methyl-2,3-diphenylquinoxaline (7)

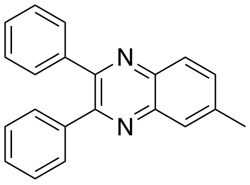

Yellow solid; Method B: 86% yield; ^{1}H NMR (400 MHz, $CDCl_3$) δ_H 8.07 (d, *J* = 8.6 Hz, 1H), 7.96 (br s, 1H), 7.61 (dd, *J* = 8.6, 1.9 Hz, 1H), 7.54 – 7.48 (m, 4H), 7.34 (d, *J* = 7.7 Hz, 6H), 2.62 (d, *J* = 1.0 Hz, 3H). ^{13}C NMR (100 MHz, $CDCl_3$) δ_C 153.5, 152.7, 141.4, 140.6, 139.9, 139.4, 132.4, 130.0, 129.96, 128.9, 128.8, 128.7, 128.3, 128.2, 22.1.

(2,3-diphenylquinoxalin-6-il)phenylmethanone (8)

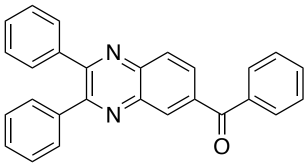

Yellow solid; Method B: 80% yield ^{1}H NMR (400 MHz, $CDCl_3$) δ_H 8.54 (dd, *J* = 1.6, 0.8 Hz, 1H), 8.36 – 8.19 (m, 2H), 7.91 (dd, *J* = 8.3, 1.3 Hz, 2H), 7.64 (t, *J* = 7.4 Hz, 1H), 7.59 – 7.50 (m, 6H), 7.42 – 7.30 (m, 6H); ^{13}C NMR (100 MHz, $CDCl_3$) δ_C 195.9, 155.3, 154.8, 143.1, 140.3, 138.8, 138.7, 138.4, 137.3, 132.9, 132.6, 130.3, 130.0, 129.99, 129.91, 129.9, 129.4, 129.3, 128.7, 128.5, 128.5.

6-nitro-(2,3-diphenyl)quinoxaline (9)

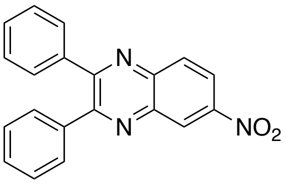

Yellow solid; Method B: 86% yield ; ^{1}H NMR (400 MHz, $CDCl_3$): δ_H 9.08 (d, *J* = 2.5 Hz, 1H), 8.53 (dd, *J* = 9.2, 2.5 Hz, 1H), 8.30 (d, *J* = 9.2 Hz, 1H), 7.62 – 7.52 (m, 5H), 7.46 – 7.35 (m, 6H); ^{13}C NMR (100 MHz, $CDCl_3$) δ 156.5, 155.8, 148.0, 143.7, 140.1, 138.24, 138.17, 130.9, 130.0, 129.97, 129.92, 129.78, 128.6, 125.8, 123.4.

6-cyano-2,3-diphenylquinoxaline (10)

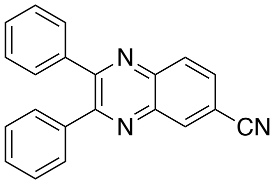

White solid; Method B: 93% yield; ^{1}H NMR (400 MHz, $CDCl_3$) δ_H 8.54 (d, *J* = 1.4 Hz, 1H), 8.25 (d, *J* = 8.6 Hz, 1H), 7.90 (dd, *J* = 8.6, 1.4 Hz, 1H), 7.54 (m, 4H), 7.45 – 7.32 (m, 6H); ^{13}C NMR (100 MHz, $CDCl_3$) δ_c 156.1, 155.5, 142.7, 140.4, 138.3, 138.3, 135.3, 130.9, 130.8, 130.0, 129.9, 129.8, 129.7, 128.6, 118.3, 113.4.

Methyl 2,3-diphenylquinoxaline-6-carboxylate (11)

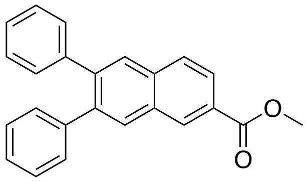

White solid; Method B: 93 % yield; ^{1}H NMR (400 MHz, $CDCl_3$) δ_H 8.91 (dd, *J* = 1.9, 0.6 Hz, 1H), 8.37 (dd, *J* = 8.7, 1.9 Hz, 1H), 8.21 (dd, *J* = 8.7, 0.6 Hz, 1H), 7.54 (m, 4H), 7.33 (m, 6H), 4.02 (s, 3H); ^{13}C NMR (100 MHz, $CDCl_3$) δ_C 166.4, 155.2, 154.5, 143.2, 140.5, 138.6, 132.0, 131.2, 129.89, 129.85, 129.5, 129.4, 129.3, 129.2, 128.4, 128.4, 52.6.

Compound 12

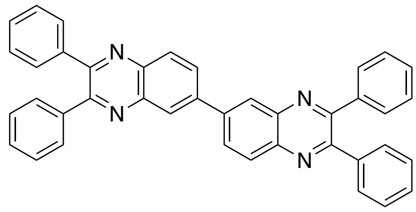

Yellow solid; Method B: 92% yield; ^{1}H NMR (400 MHz, $CDCl_3$) δ_H 8.61 (d, *J* = 1.9 Hz, 2H), 8.33 (d, *J* = 8.7 Hz, 2H), 8.24 (dd, *J* = 8.7, 1.9 Hz, 2H), 7.61 – 7.53 (m, 8H), 7.42 – 7.32 (m, 12H). ^{13}C NMR (100 MHz, $CDCl_3$) δ_c 154.3, 153.9, 141.5, 141.4, 141.0, 139.03, 139.02, 130.1, 130.0, 129.7, 129.2, 129.1, 128.5, 127.6.

Compound 13

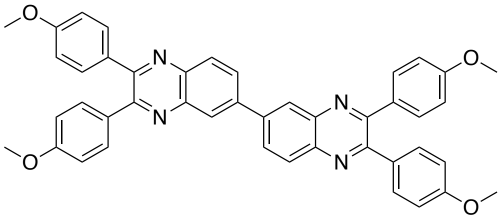

Yellow solid; Method B: 94% yield; ^{1}H NMR (400 MHz, $CDCl_3$) δ_H 8.56 (br s, 2H), 8.28 (d, *J* = 8.7 Hz, 1H), 8.19 (d, *J* = 8.7 Hz, 1H), 7.55 (d, *J* = 7.7 Hz, 8H), 6.90 (d, *J* = 7.7 Hz, 8H), 3.85 (s, 12H); ^{13}C NMR (100 MHz, $CDCl_3$) δ_c 160.4, 153.6, 153.2, 141.1, 141.0, 140.6, 131.4, 129.6, 129.2, 127.1, 113.9, 55.4.

Compound 14

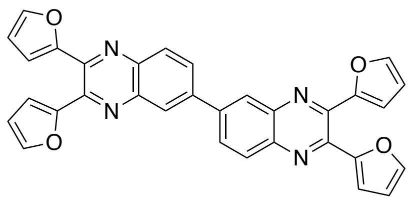

Yellow solid; Method B: 93% yield; ^{1}H NMR (400 MHz, $CDCl_3$) δ_H 8.52 (d, *J* = 1.8 Hz, 2H), 8.26 (d, *J* = 8.7 Hz, 2H), 8.18 (dd, *J* = 8.7, 1.8 Hz, 2H), 7.65 (s, 4H), 6.73 (dd, *J* = 9.6, 3.4 Hz, 4H), 6.59 (dt, *J* = 3.4, 1.9 Hz, 4H). ^{13}C NMR (100 MHz, $CDCl_3$) δ_C 150.9, 144.6, 144.5, 143.4, 142.9, 141.5, 140.9, 140.5, 129.99, 129.97, 127.4, 113.5, 113.5, 112.2.

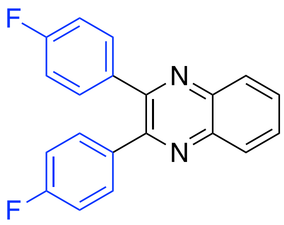

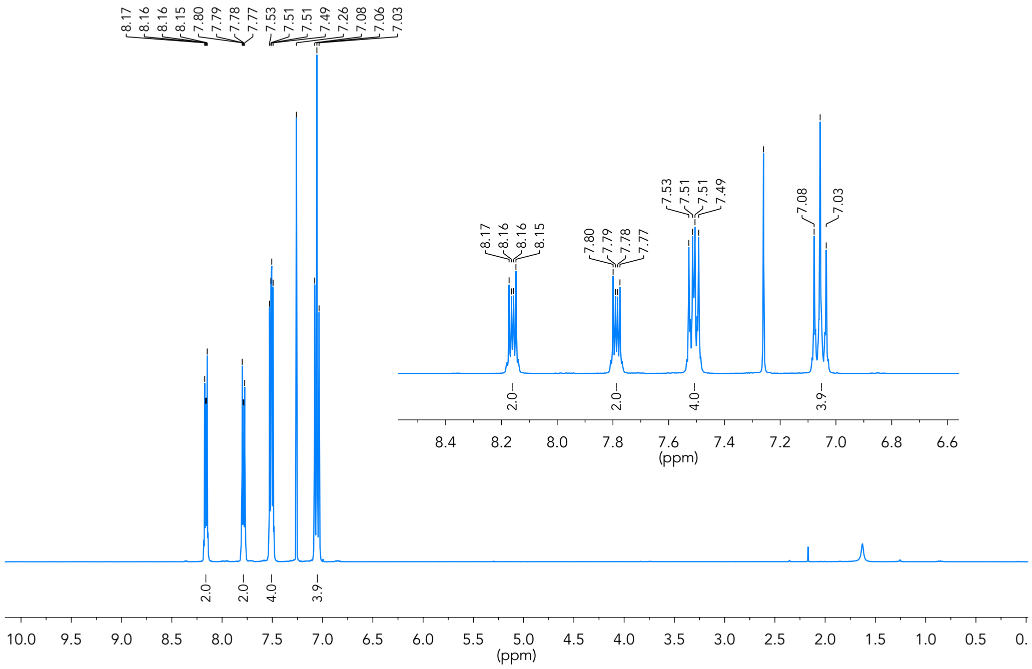

Figure SI3. ^{1}H NMR (400 MHz, $CDCl_3$) of quinoxaline **2**

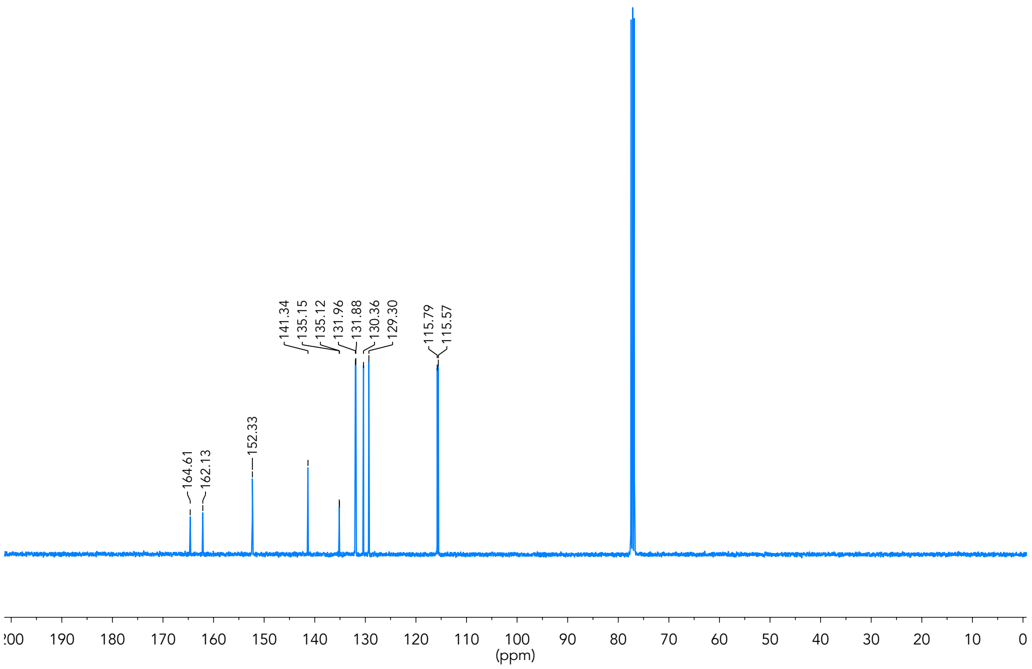

Figure SI4. ^{13}C NMR (100 MHz, $CDCl_3$) of quinoxaline **2**

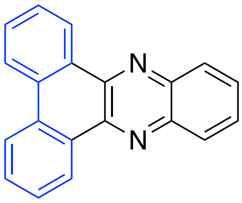

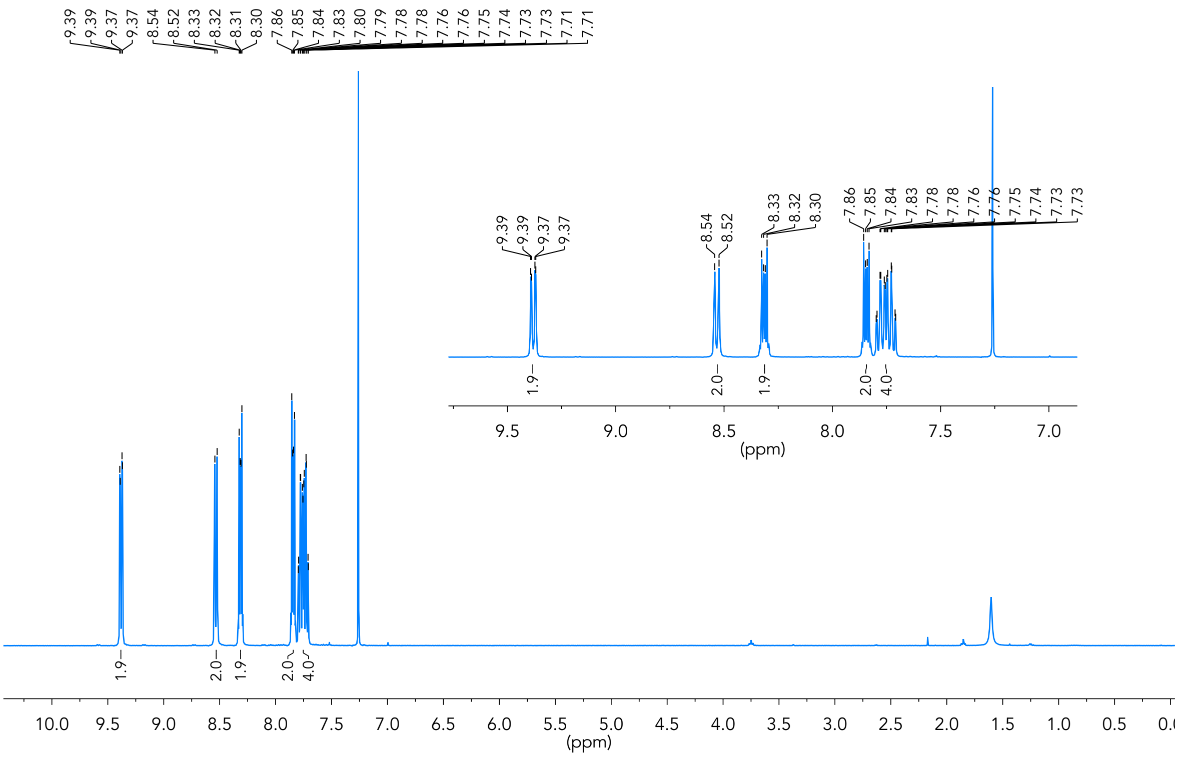

Figure SI5. ^{1}H NMR (400 MHz, $CDCl_3$) of quinoxaline **3**

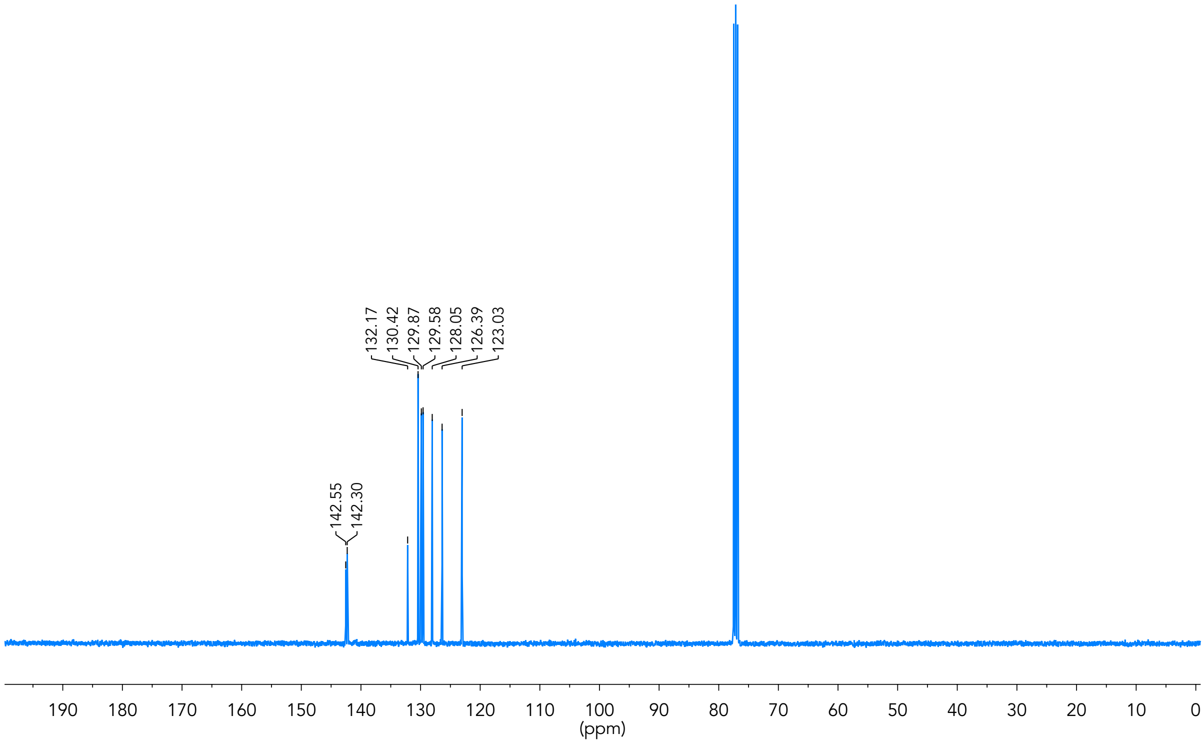

Figure SI6. ^{13}C NMR (100 MHz, $CDCl_3$) of quinoxaline **3**

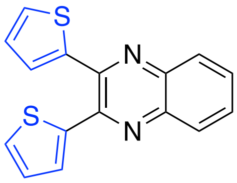

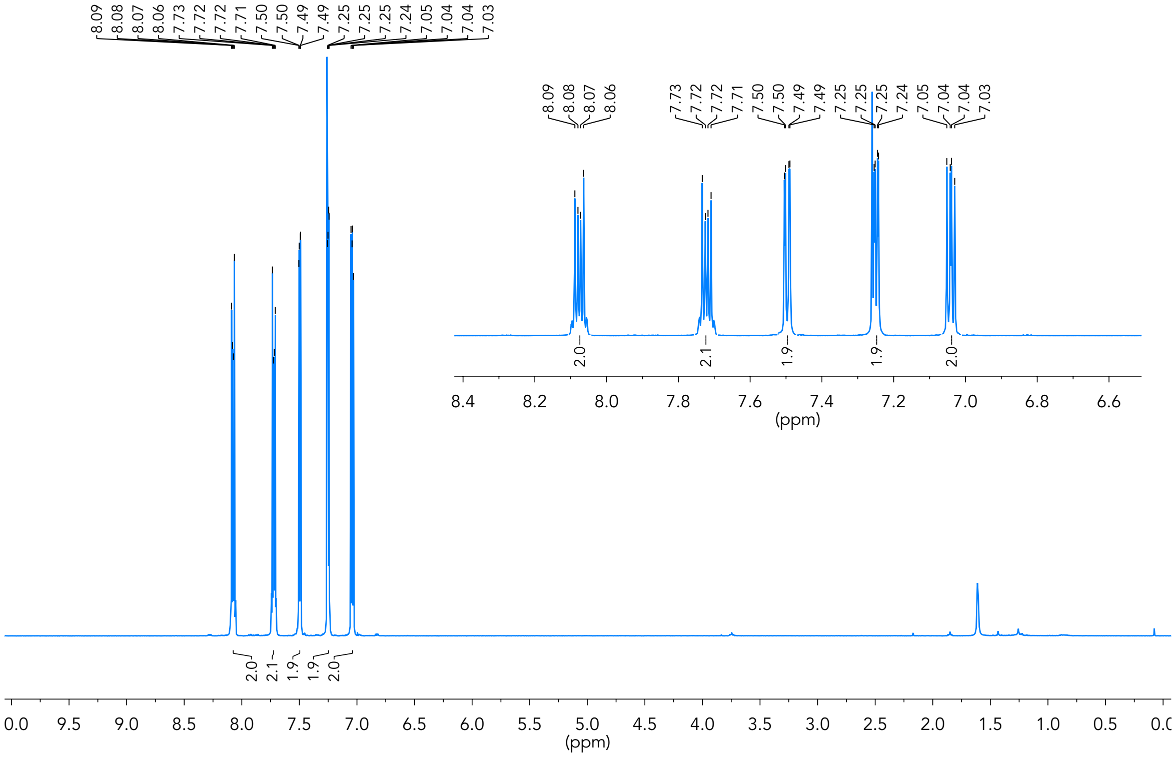

Figure SI7. ^{1}H NMR (400 MHz, $CDCl_3$) of quinoxaline **4**

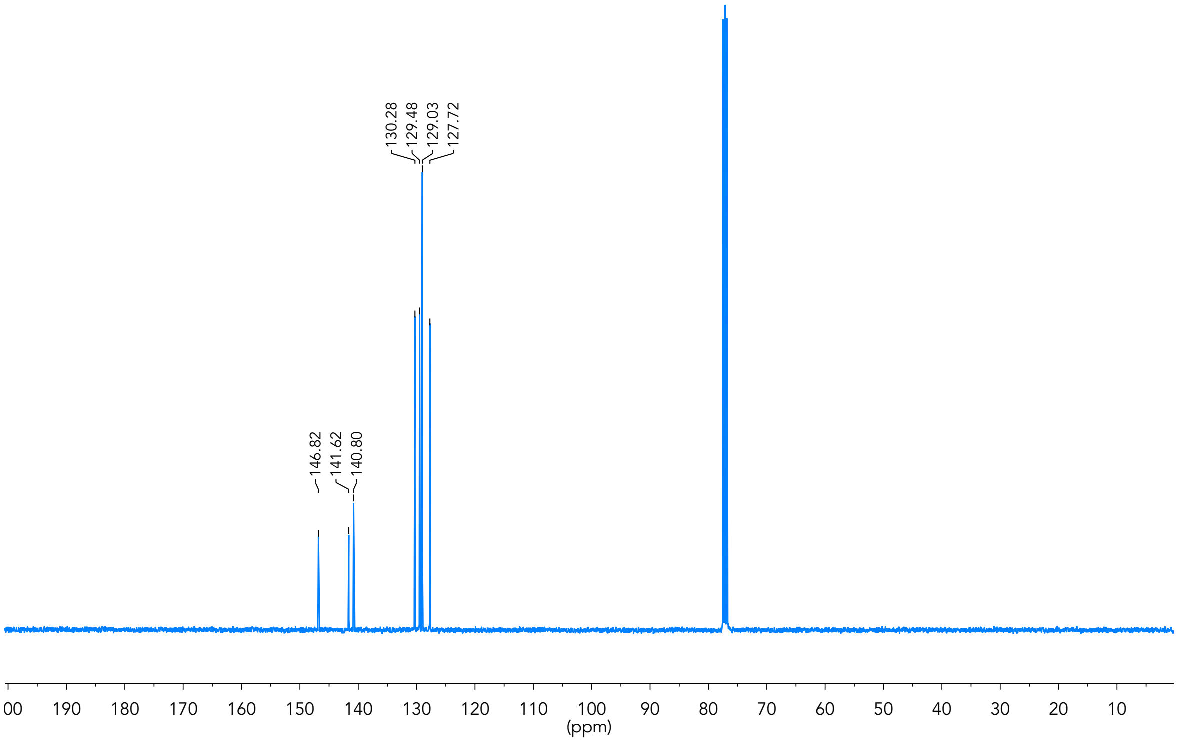

Figure SI8. ^{13}C NMR (100 MHz, $CDCl_3$) of quinoxaline **4**

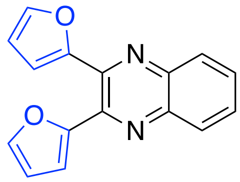

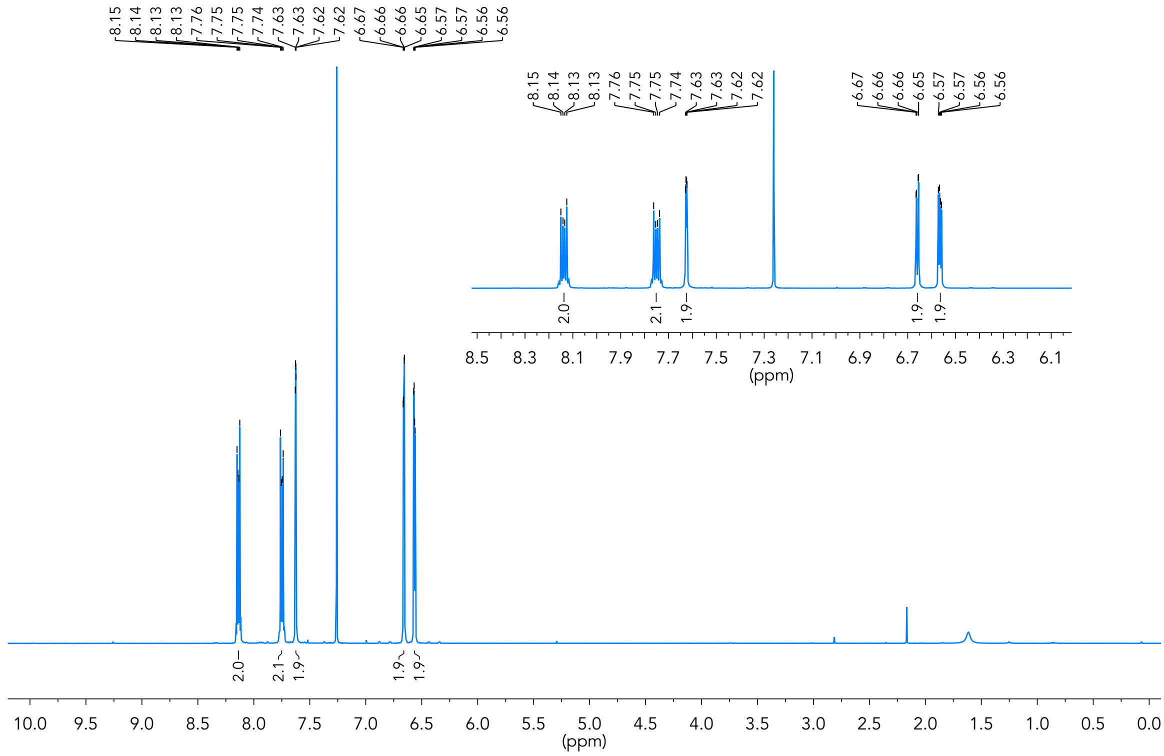

Figure SI9. ^{1}H NMR (400 MHz, $CDCl_3$) of quinoxaline **5**

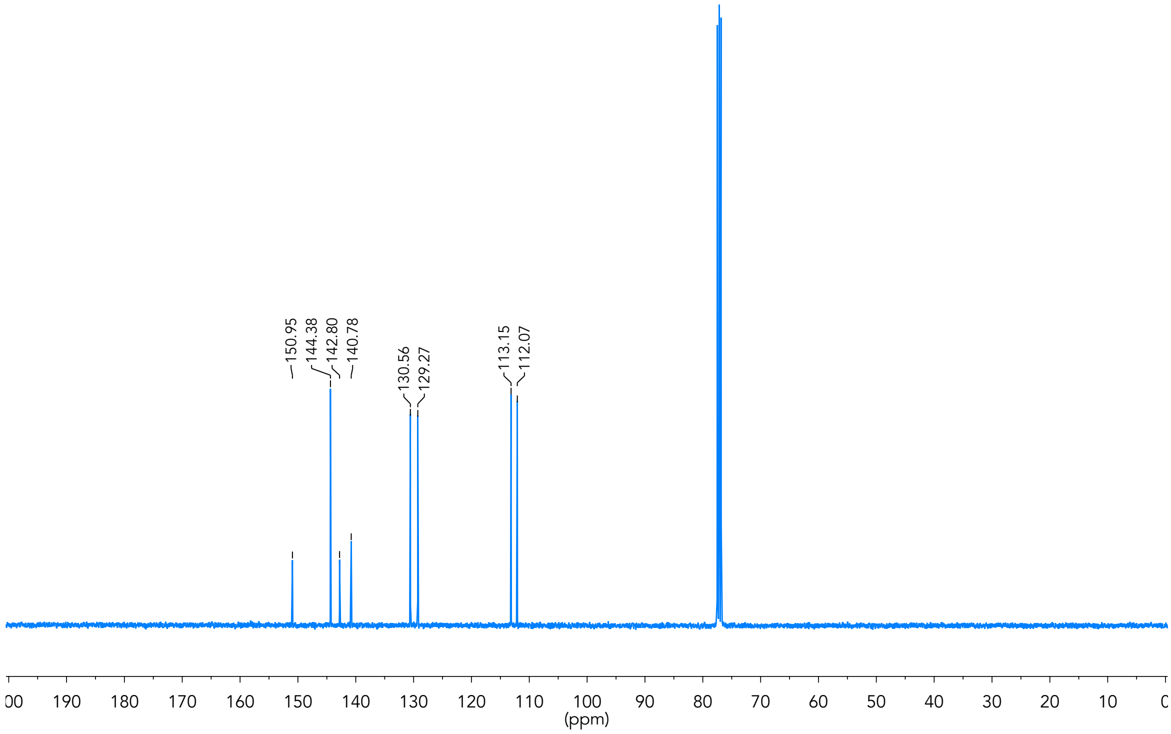

Figure SI10. ^{13}C NMR (100 MHz, $CDCl_3$) of quinoxaline **5**

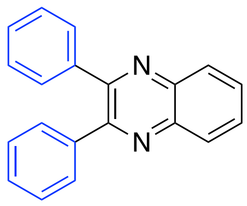

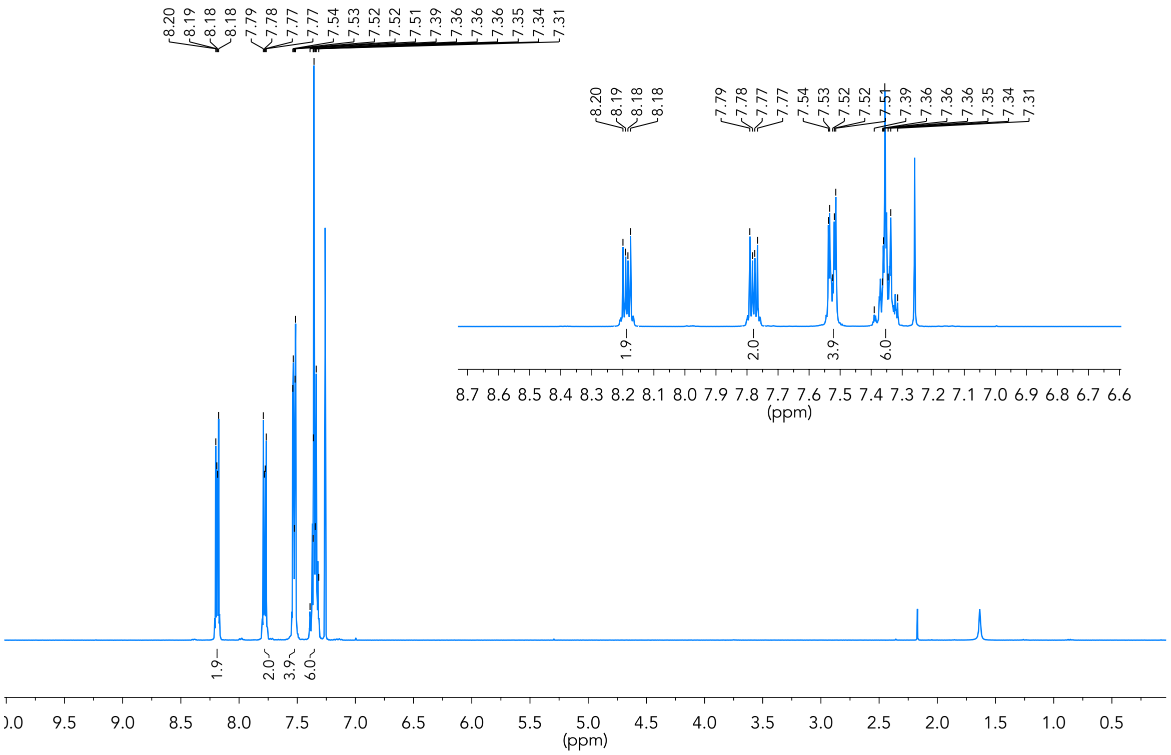

Figure SI11. ^{1}H NMR (400 MHz, $CDCl_3$) of quinoxaline **6**

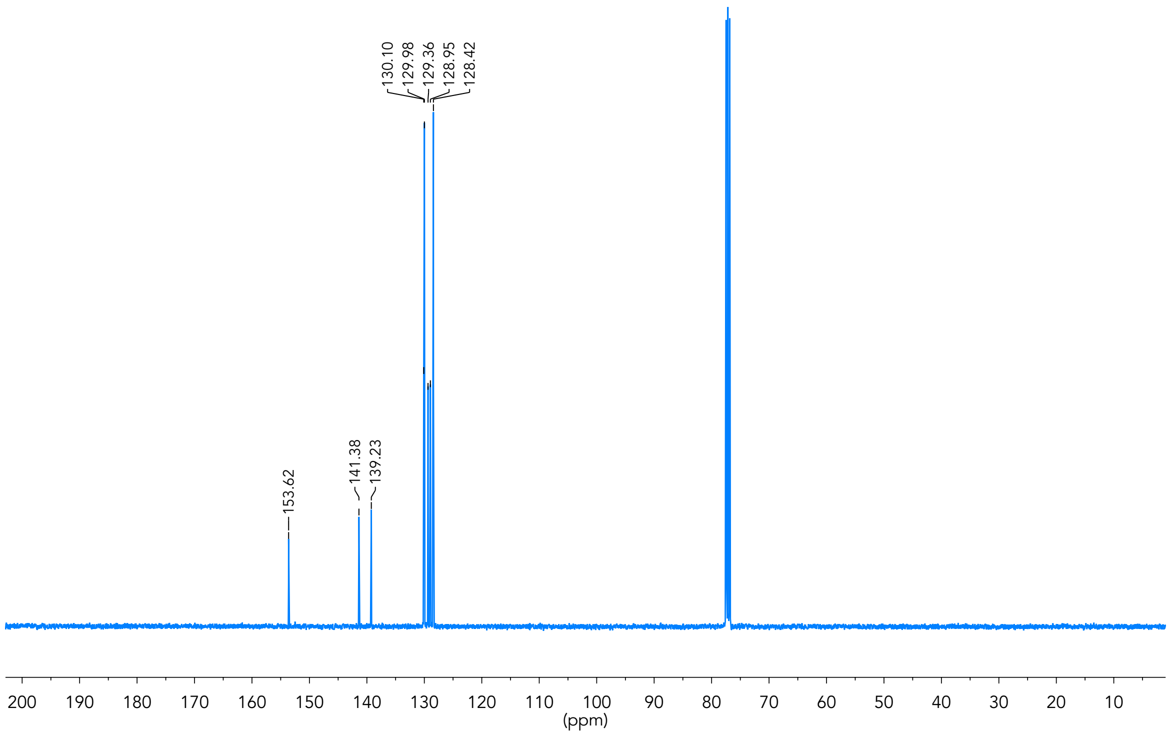

Figure SI12. ^{13}C NMR (100 MHz, $CDCl_3$) of quinoxaline **6**

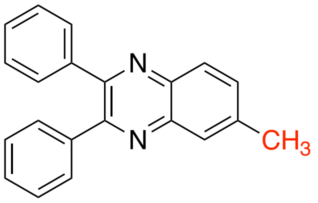

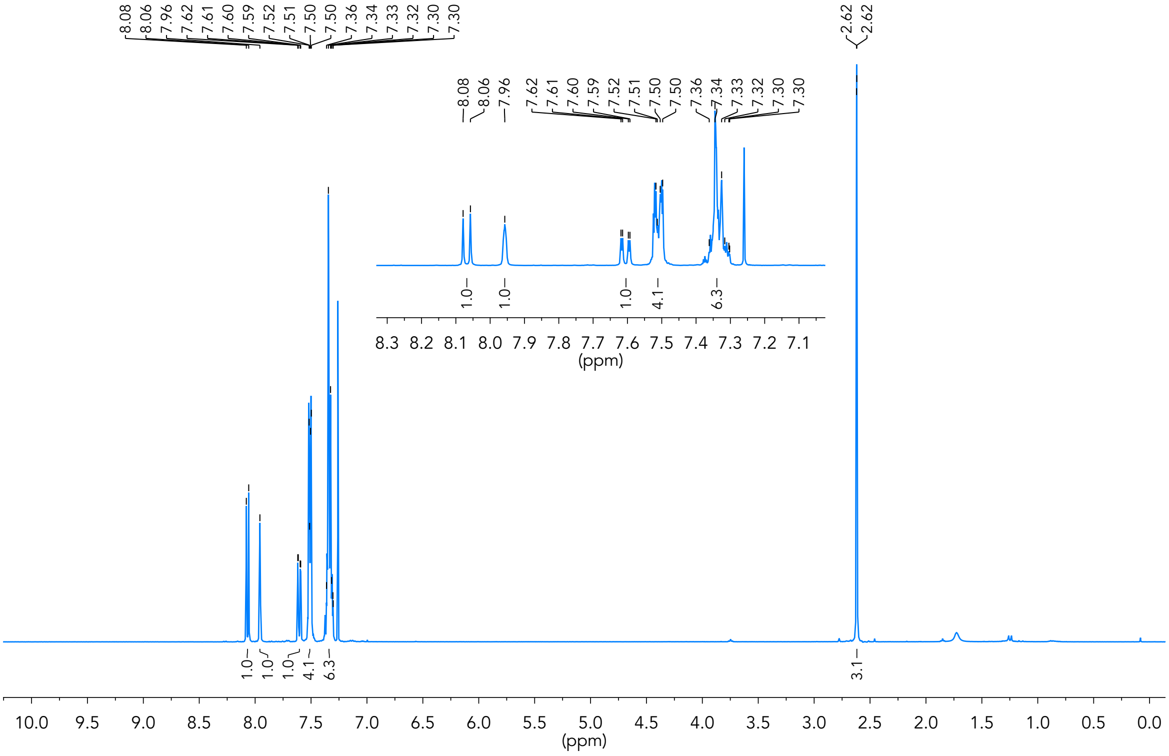

Figure SI13. ^{1}H NMR (400 MHz, $CDCl_3$) of quinoxaline **7**

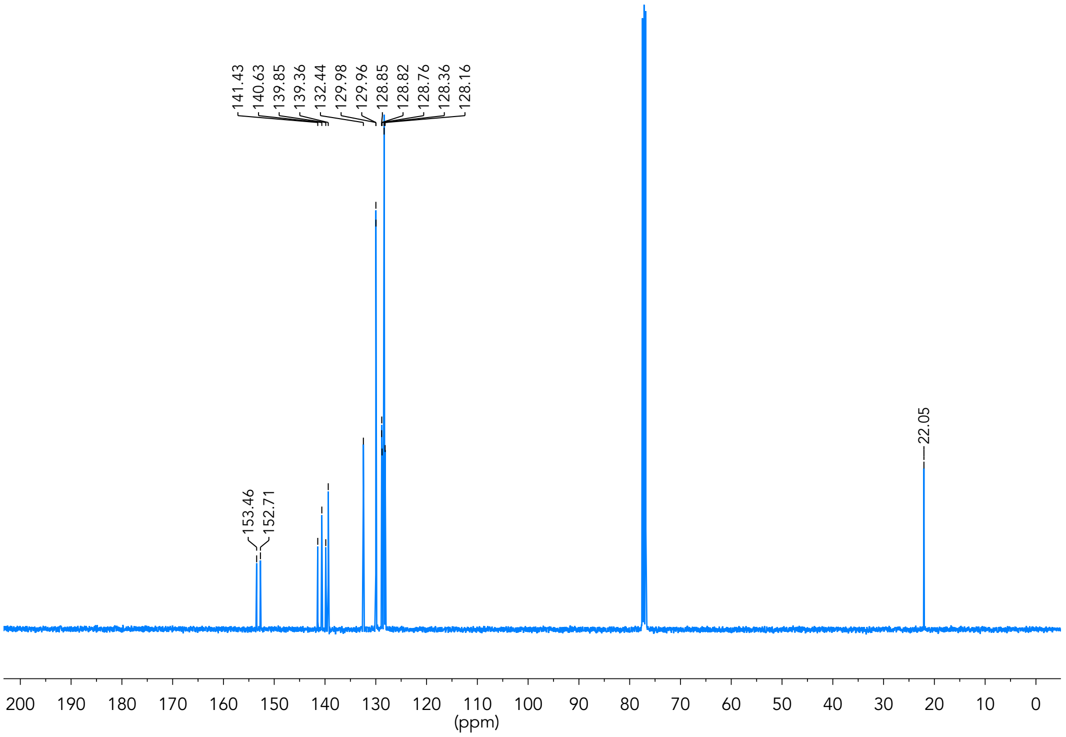

Figure SI14. ^{13}C NMR (100 MHz, $CDCl_3$) of quinoxaline **7**

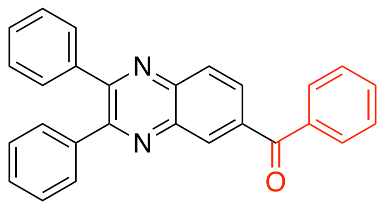

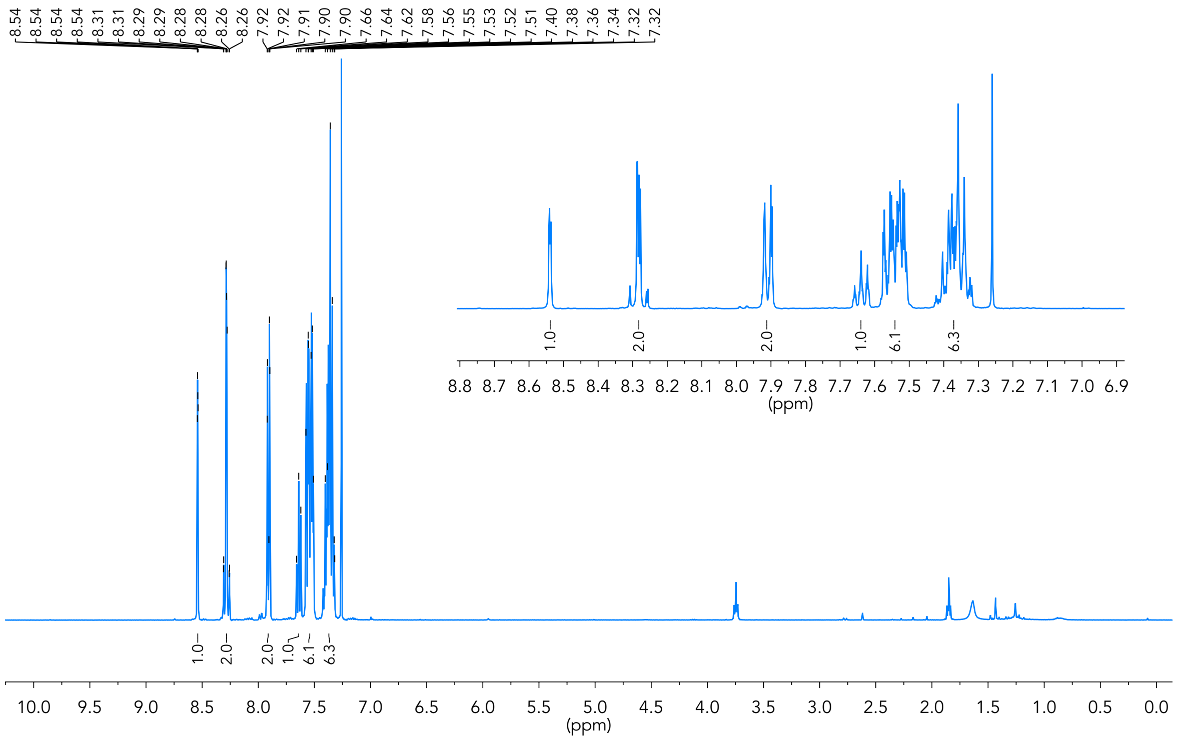

Figure SI15. ^{1}H NMR (400 MHz, $CDCl_3$) of quinoxaline **8**

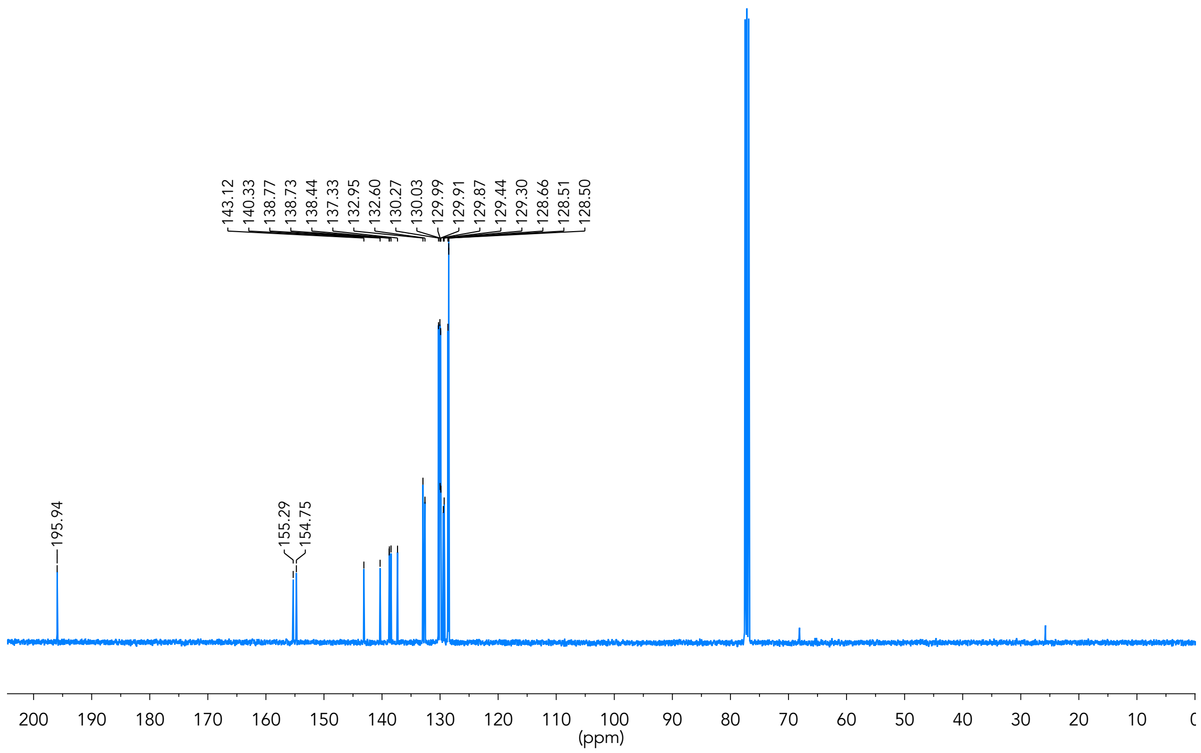

Figure SI16. ^{13}C NMR (100 MHz, $CDCl_3$) of quinoxaline **8**

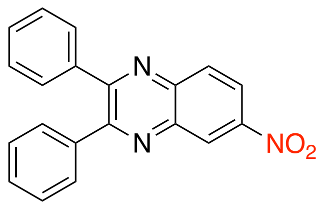

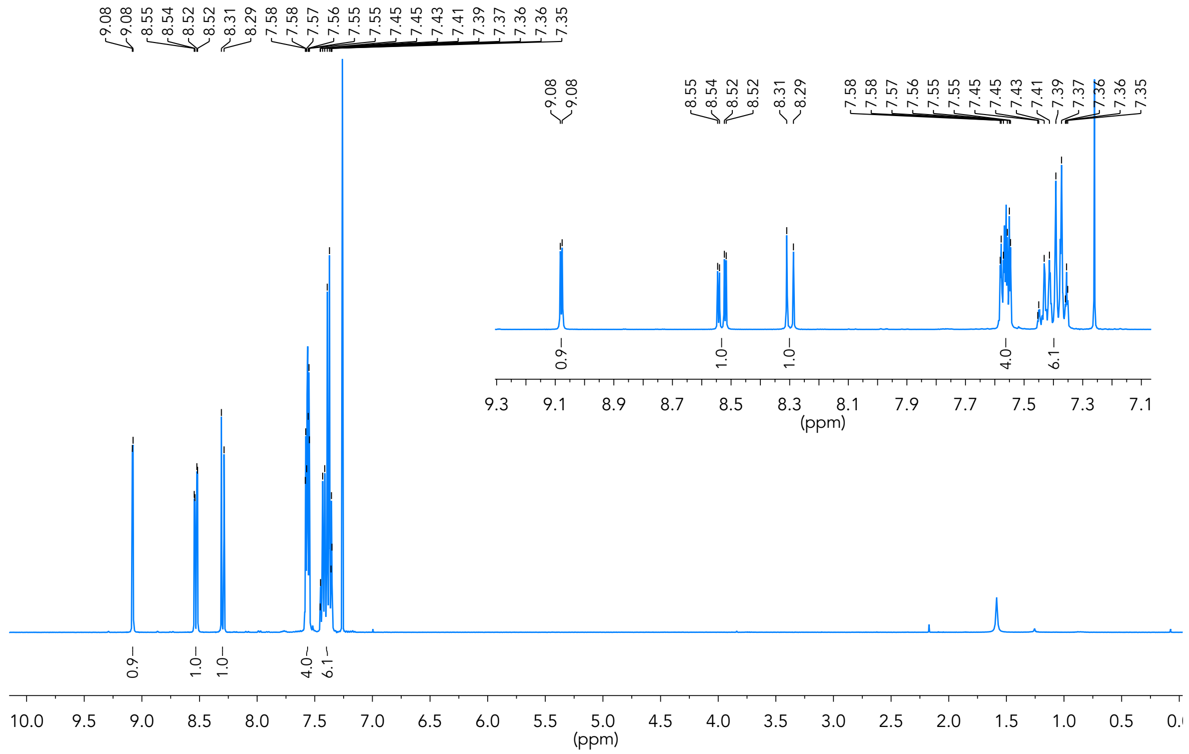

Figure SI17. ^{1}H NMR (400 MHz, $CDCl_3$) of quinoxaline **9**

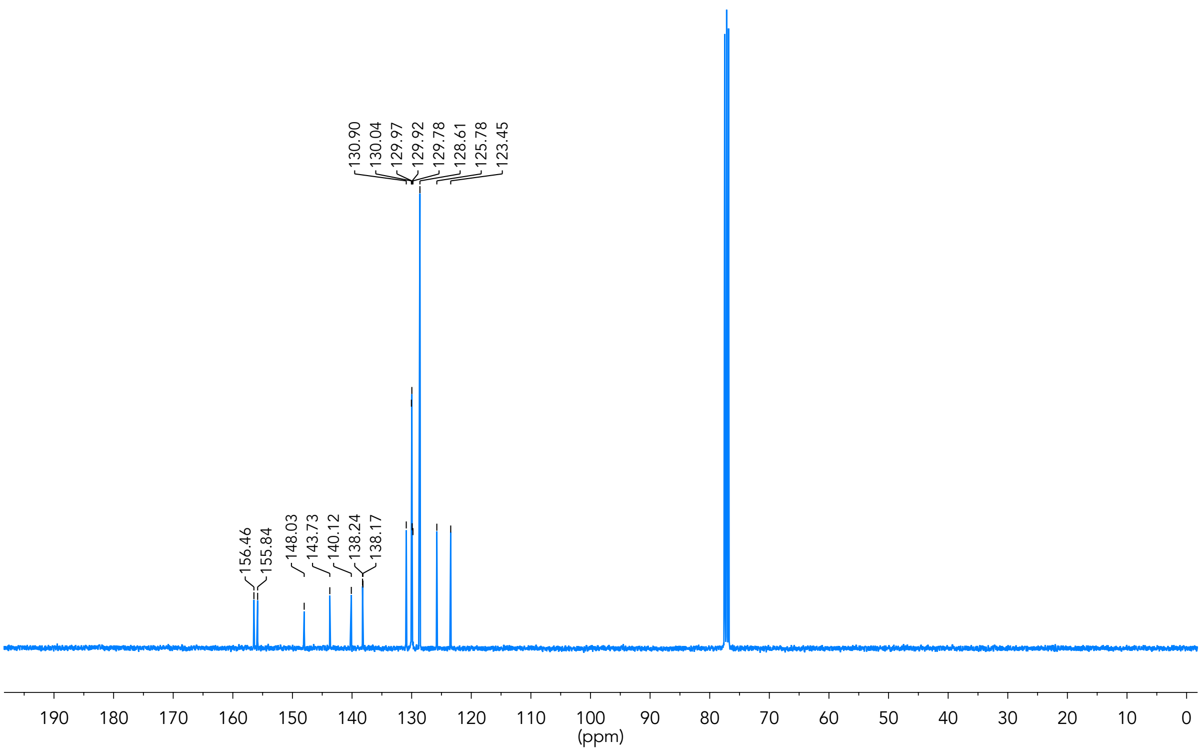

Figure SI18. ^{13}C NMR (100 MHz, $CDCl_3$) of quinoxaline **9**

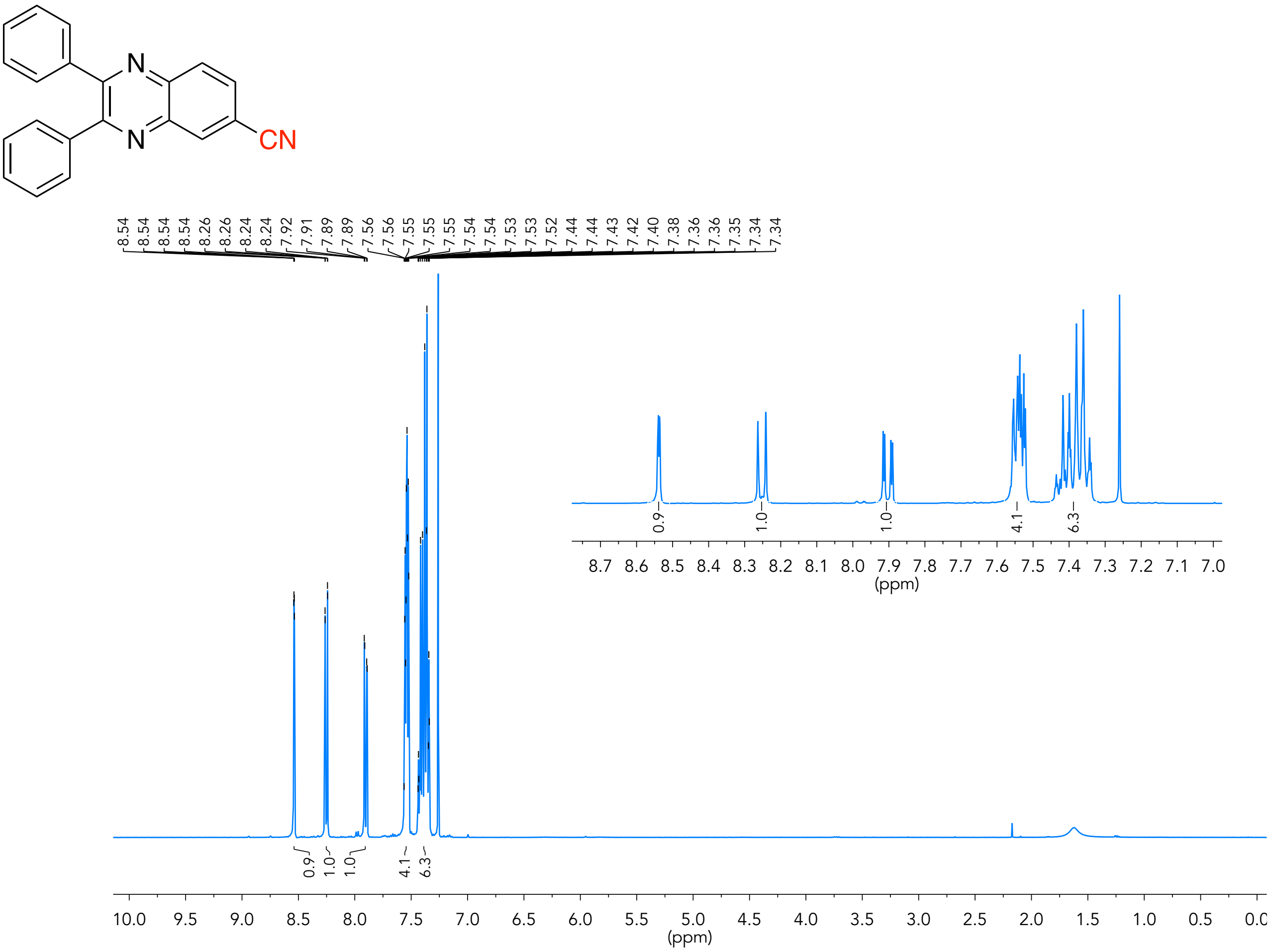

Figure SI19. ^{1}H NMR (400 MHz, $CDCl_3$) of quinoxaline **10**

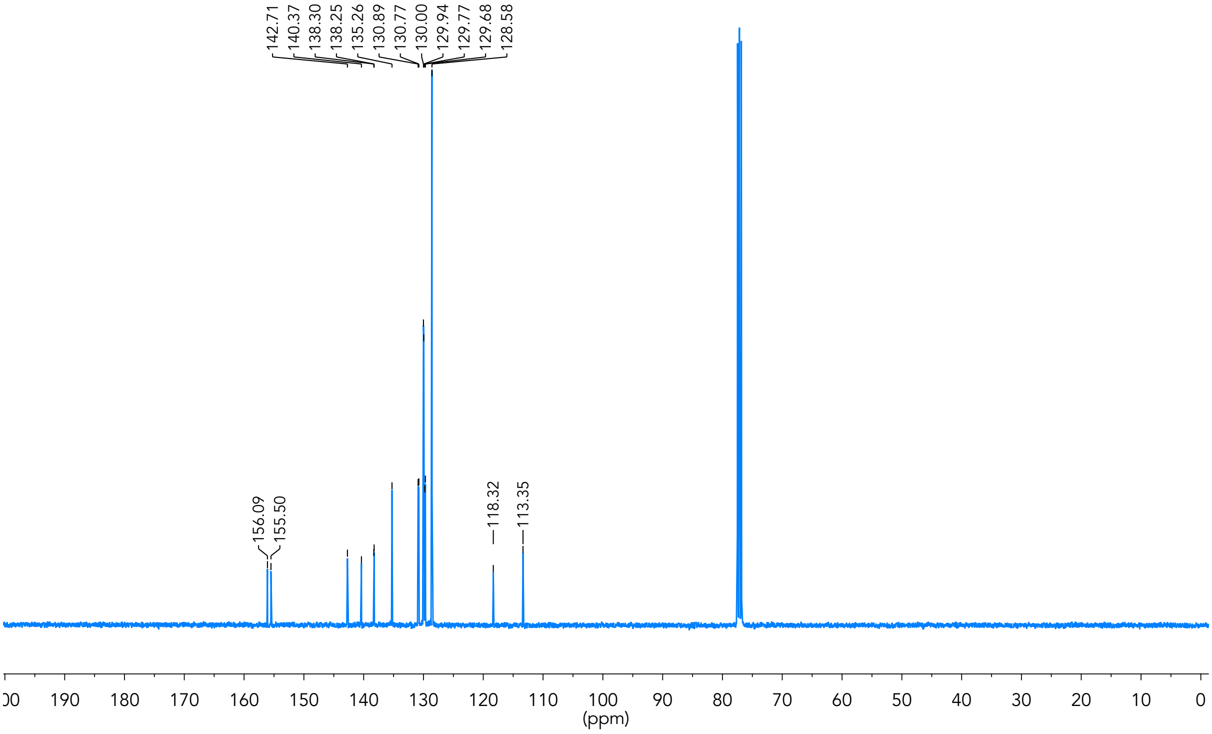

Figure SI20. ^{13}C NMR (100 MHz, $CDCl_3$) of quinoxaline **10**

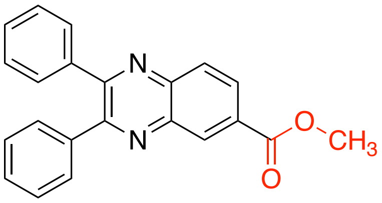

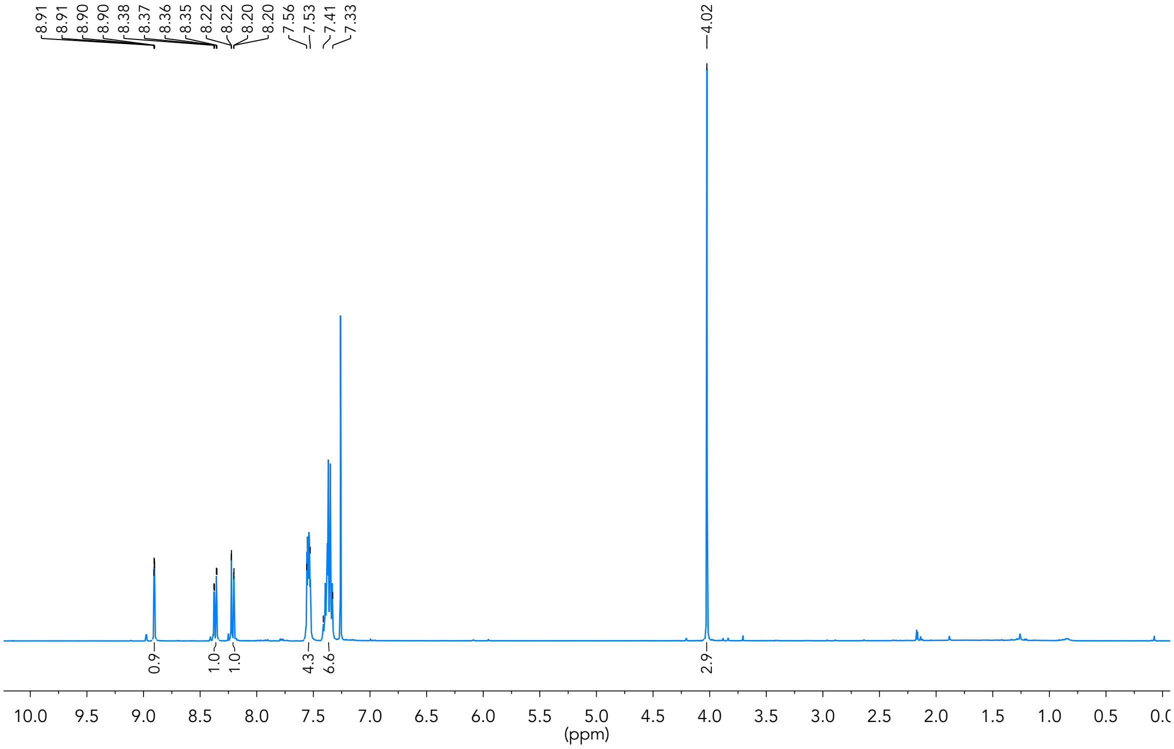

Figure SI21. ^{1}H NMR (400 MHz, $CDCl_3$) of quinoxaline **11**

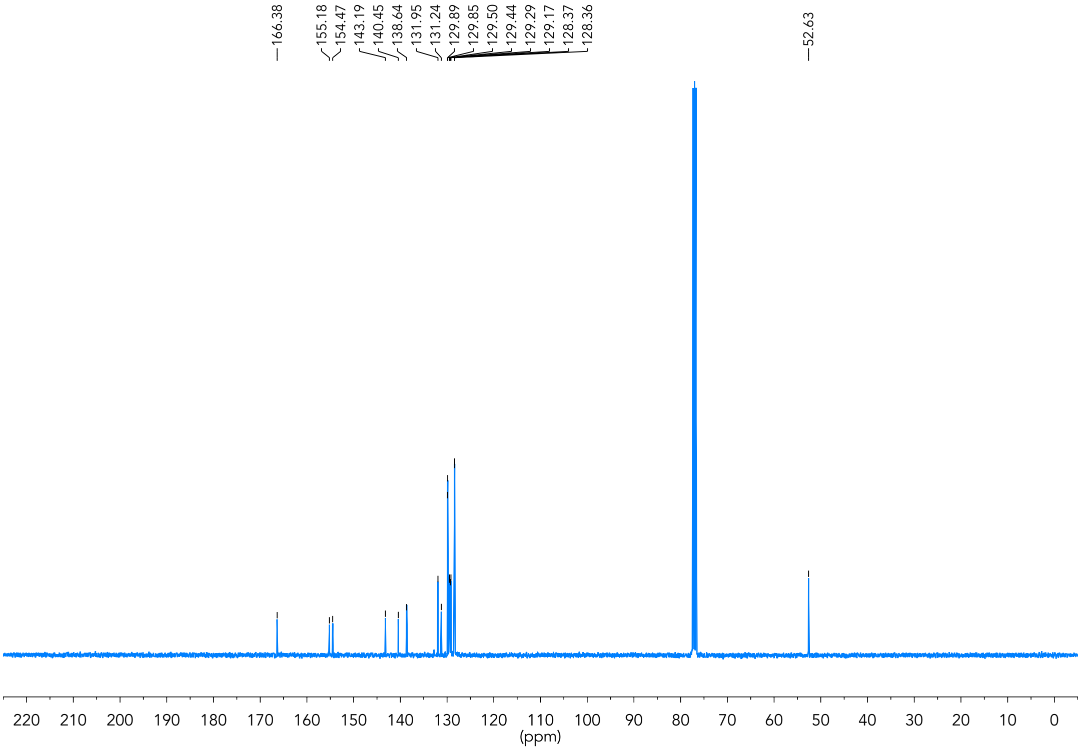

Figure SI22. ^{13}C NMR (100 MHz, $CDCl_3$) of quinoxaline **11**

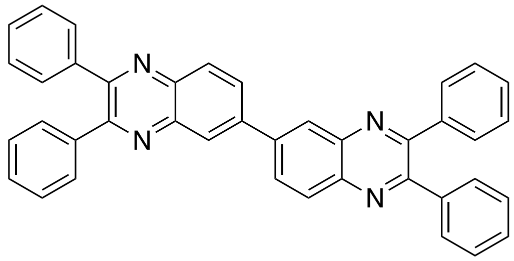

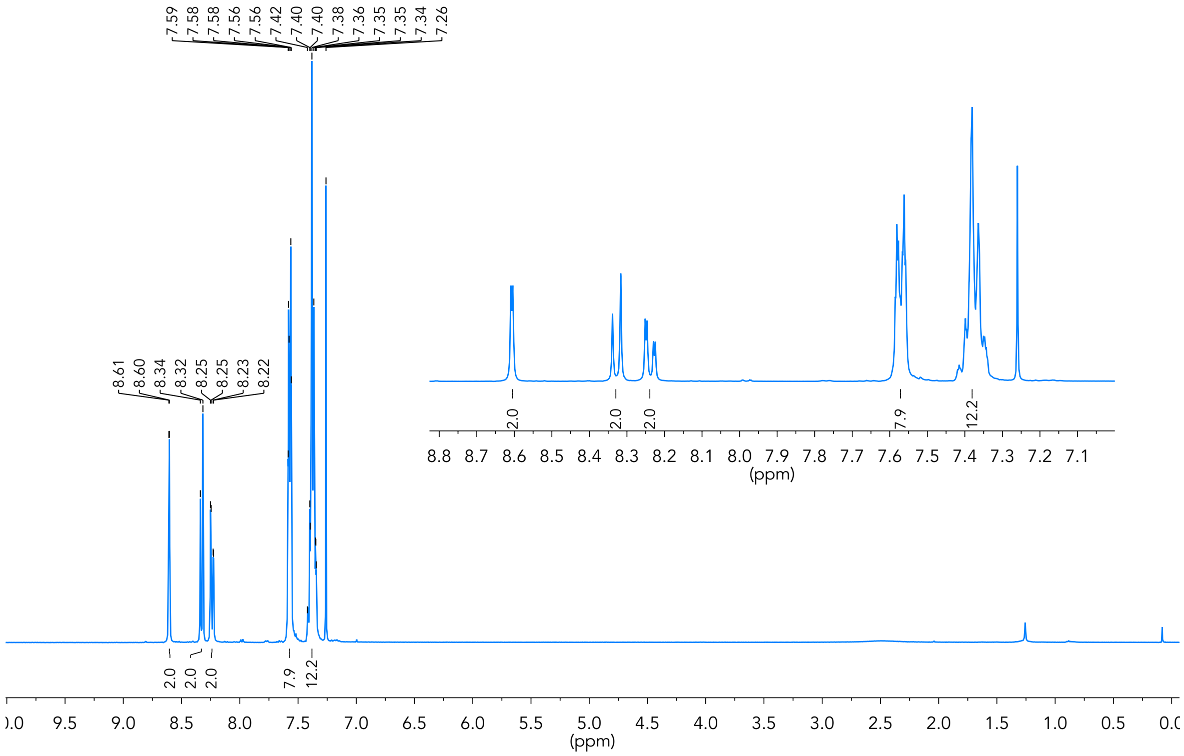

Figure SI23. ^{1}H NMR (400 MHz, $CDCl_3$) of quinoxaline **12**

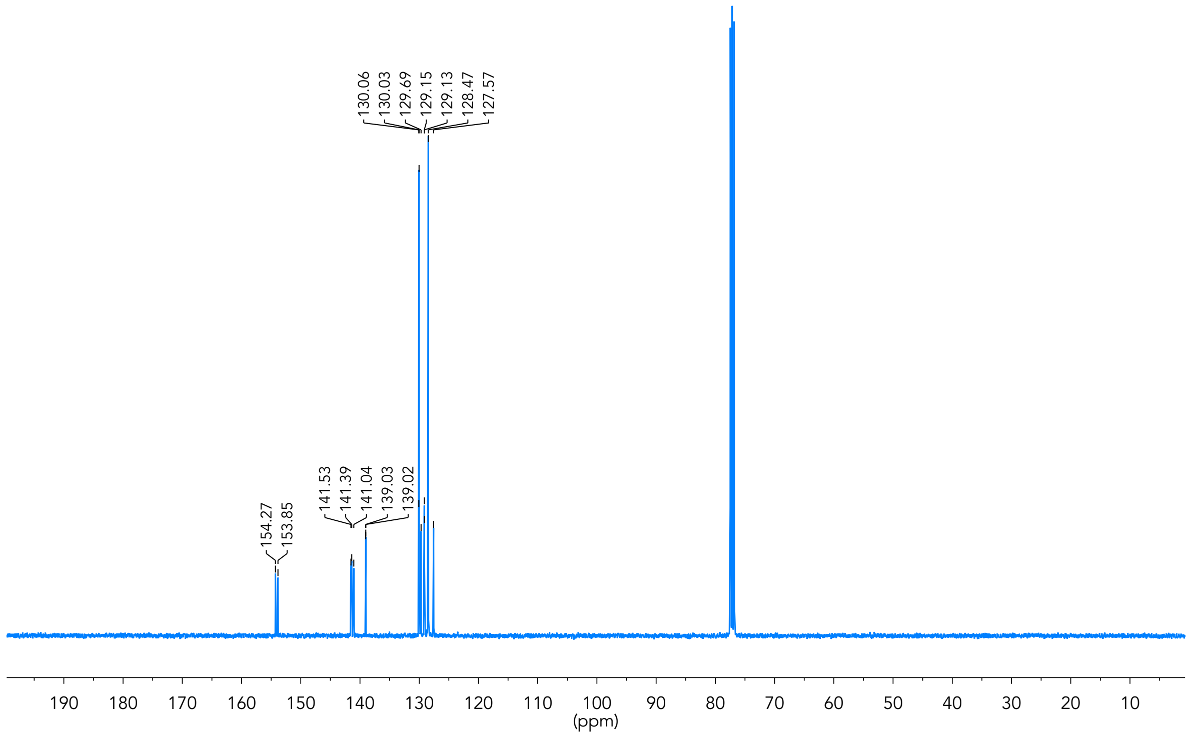

Figure SI24. ^{13}C NMR (100 MHz, $CDCl_3$) of quinoxaline **12**

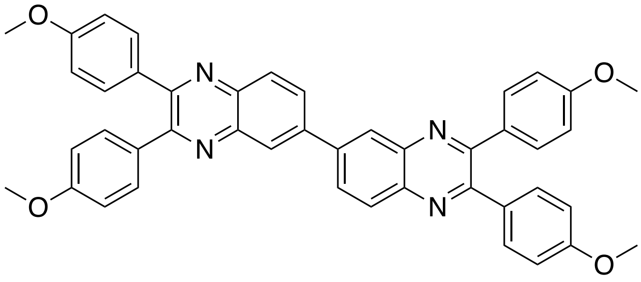

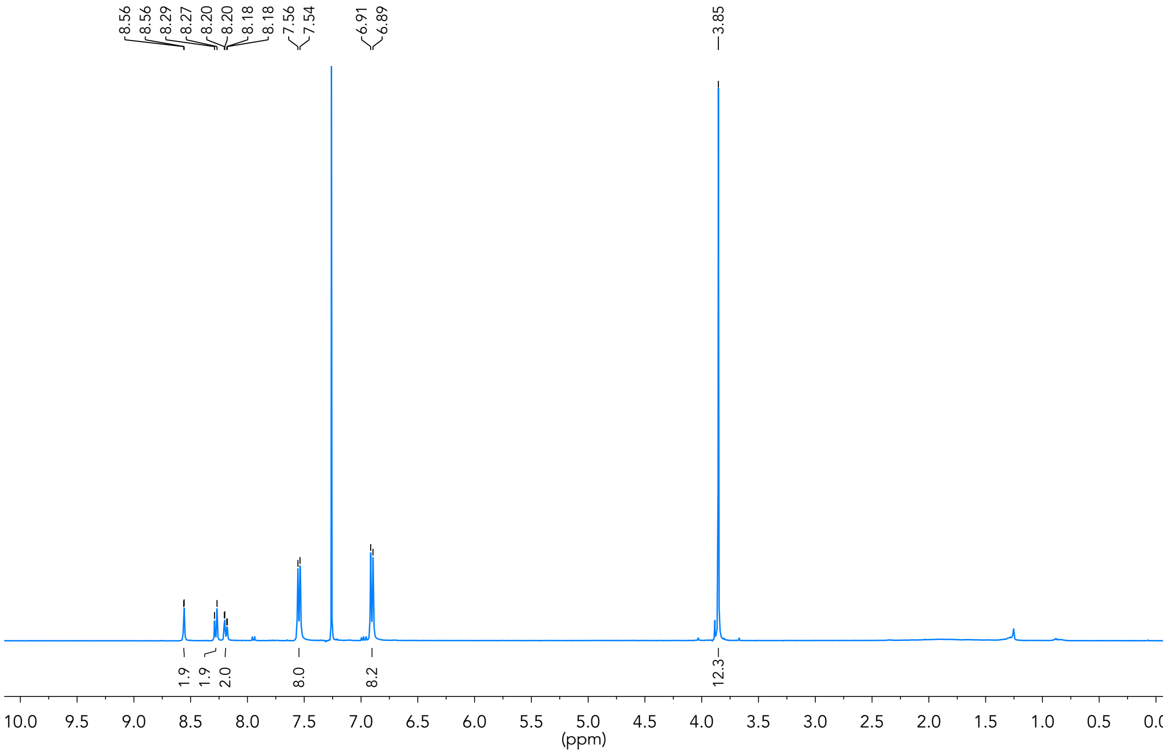

Figure SI25. ^{1}H NMR (400 MHz, $CDCl_3$) of quinoxaline **13**

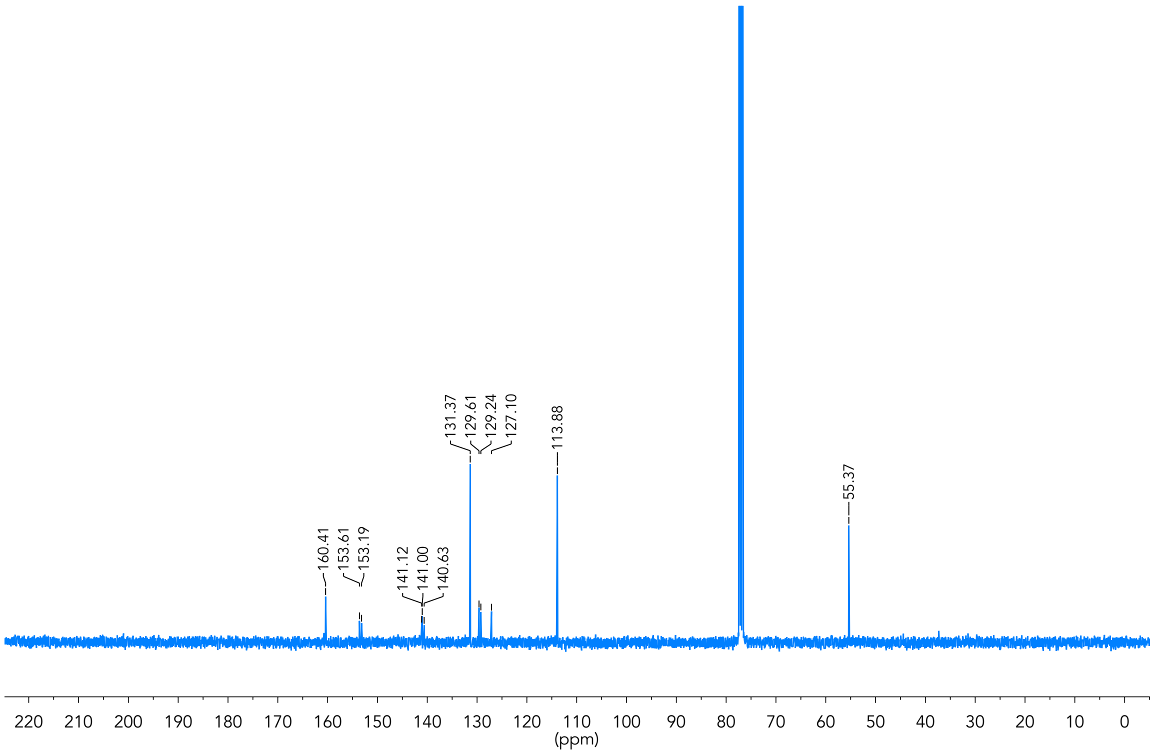

Figure SI26. ^{13}C NMR (100 MHz, $CDCl_3$) of quinoxaline **13**

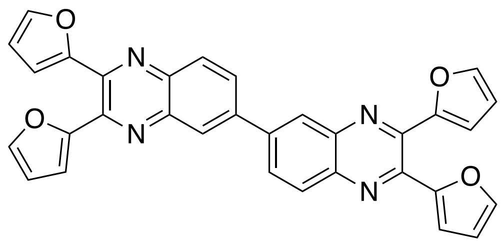

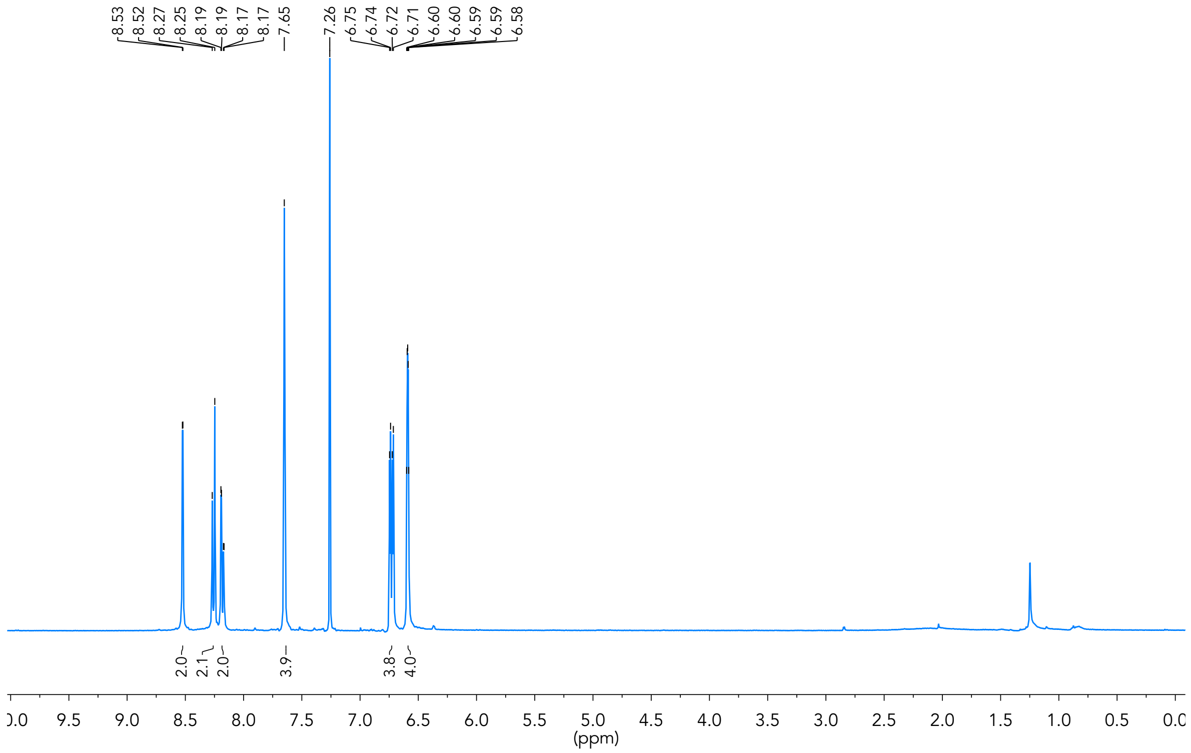

Figure SI27. ^{1}H NMR (400 MHz, $CDCl_3$) of quinoxaline **14**

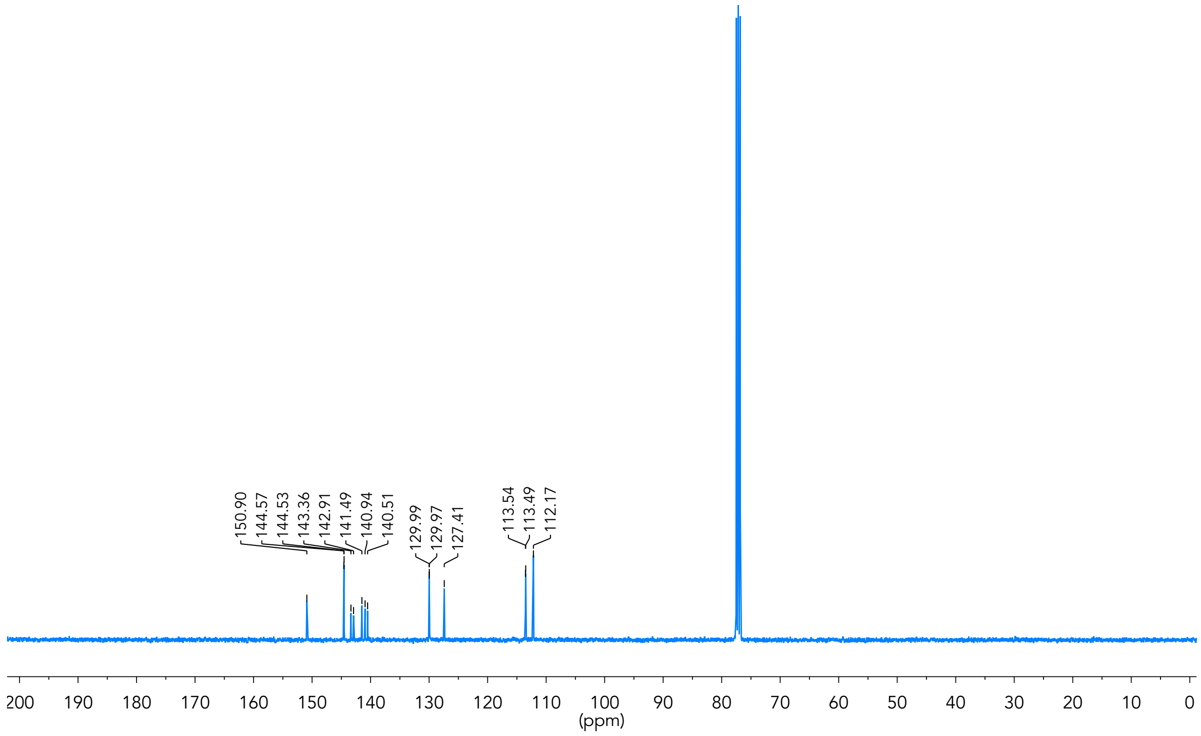

Figure SI28. ^{13}C NMR (100 MHz, $CDCl_3$) of quinoxaline **14**

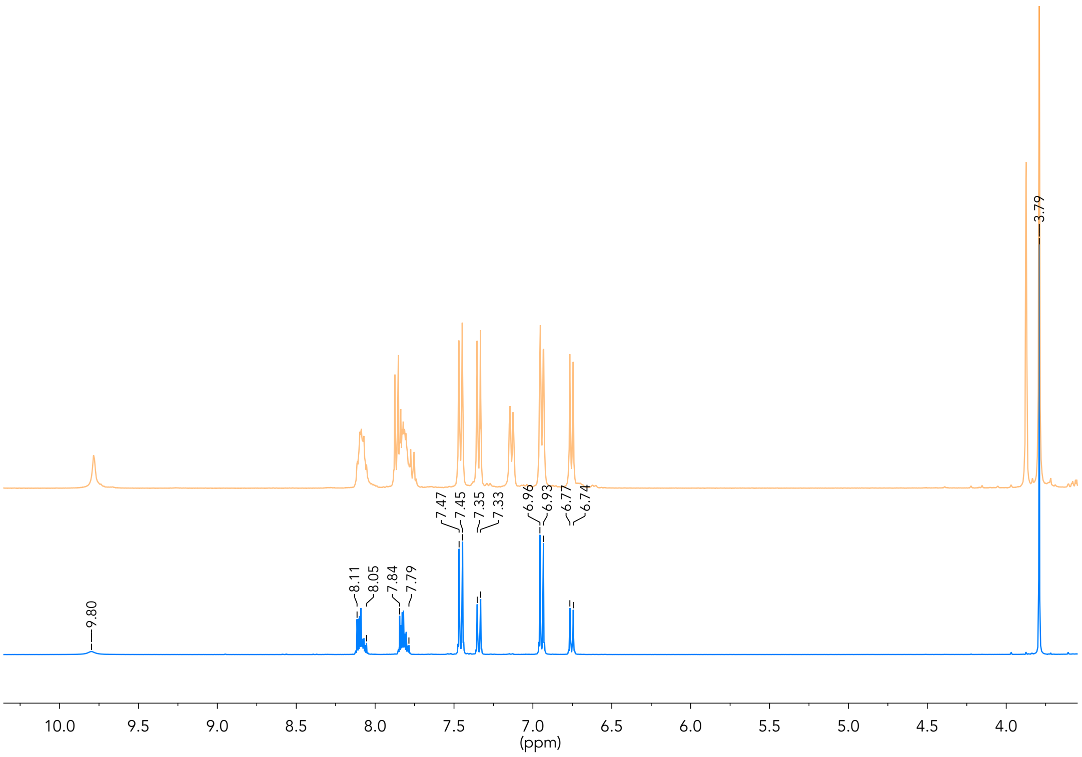

Figure SI29. ^{1}H NMR (400 MHz, DMSO-d_6) of crude reaction product between o-PDA dihydrochloride and 4,4´-dimethoxybenzil: method A (Bottom), method B (Top).

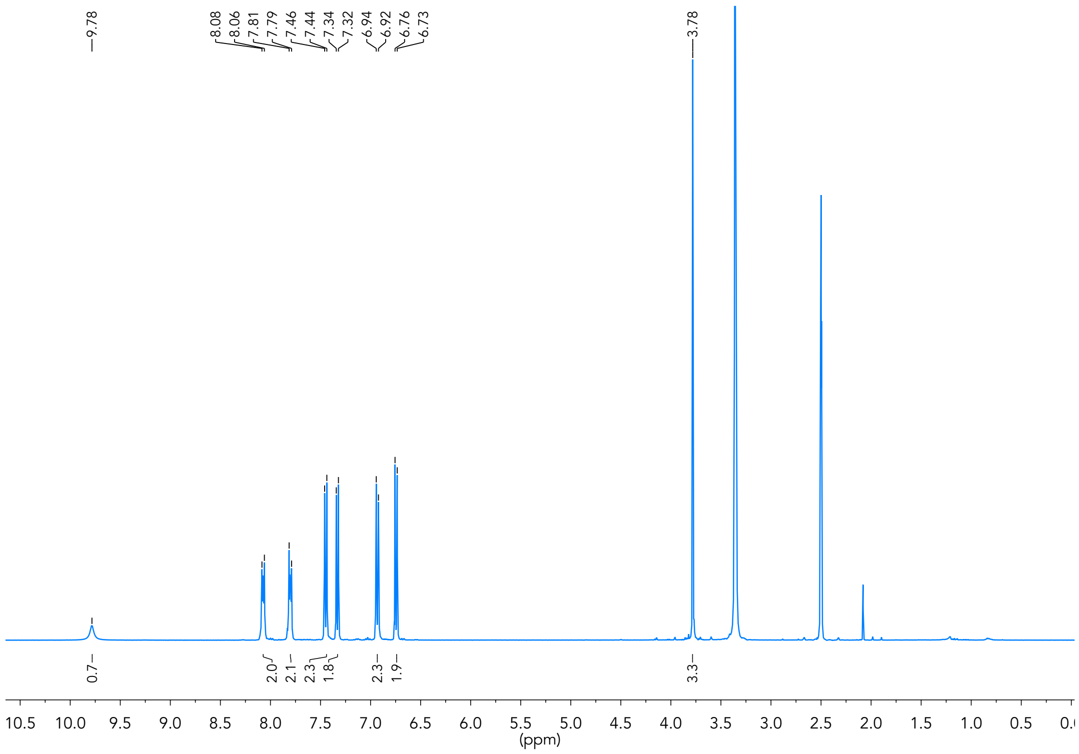

Figure SI30. ^{1}H NMR (400 MHz, DMSO-d_6) of quinoxaline **1a**

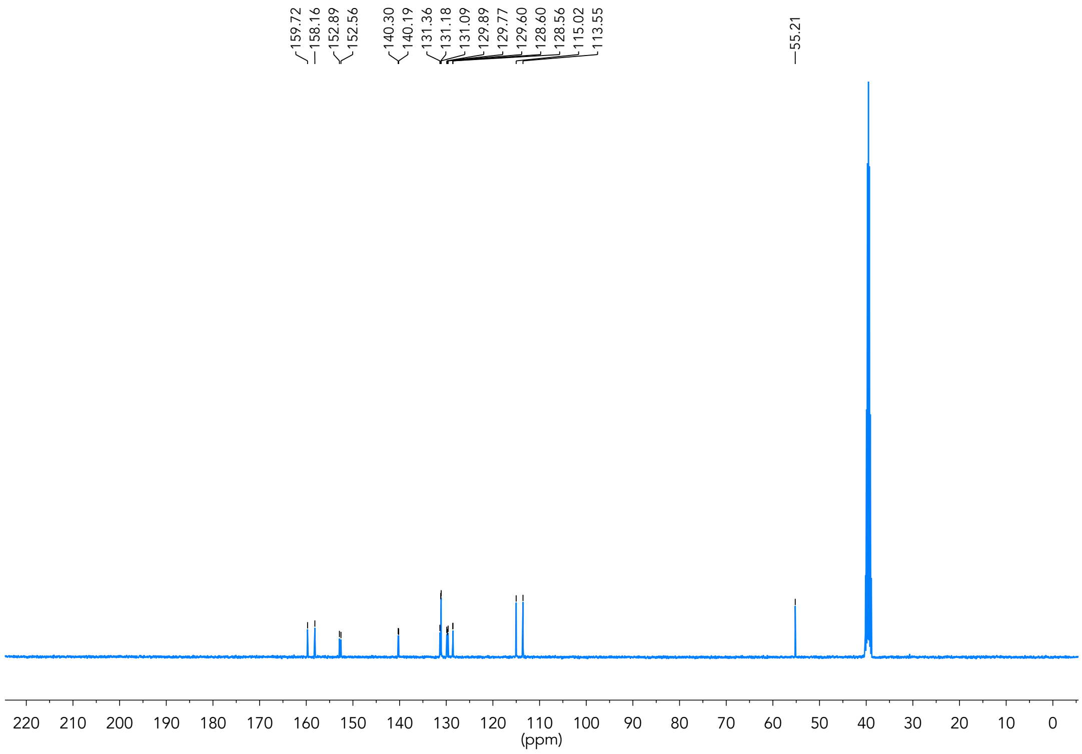

Figure SI31. ^{13}C NMR (100 MHz, $CDCl_3$) of quinoxaline **1a**

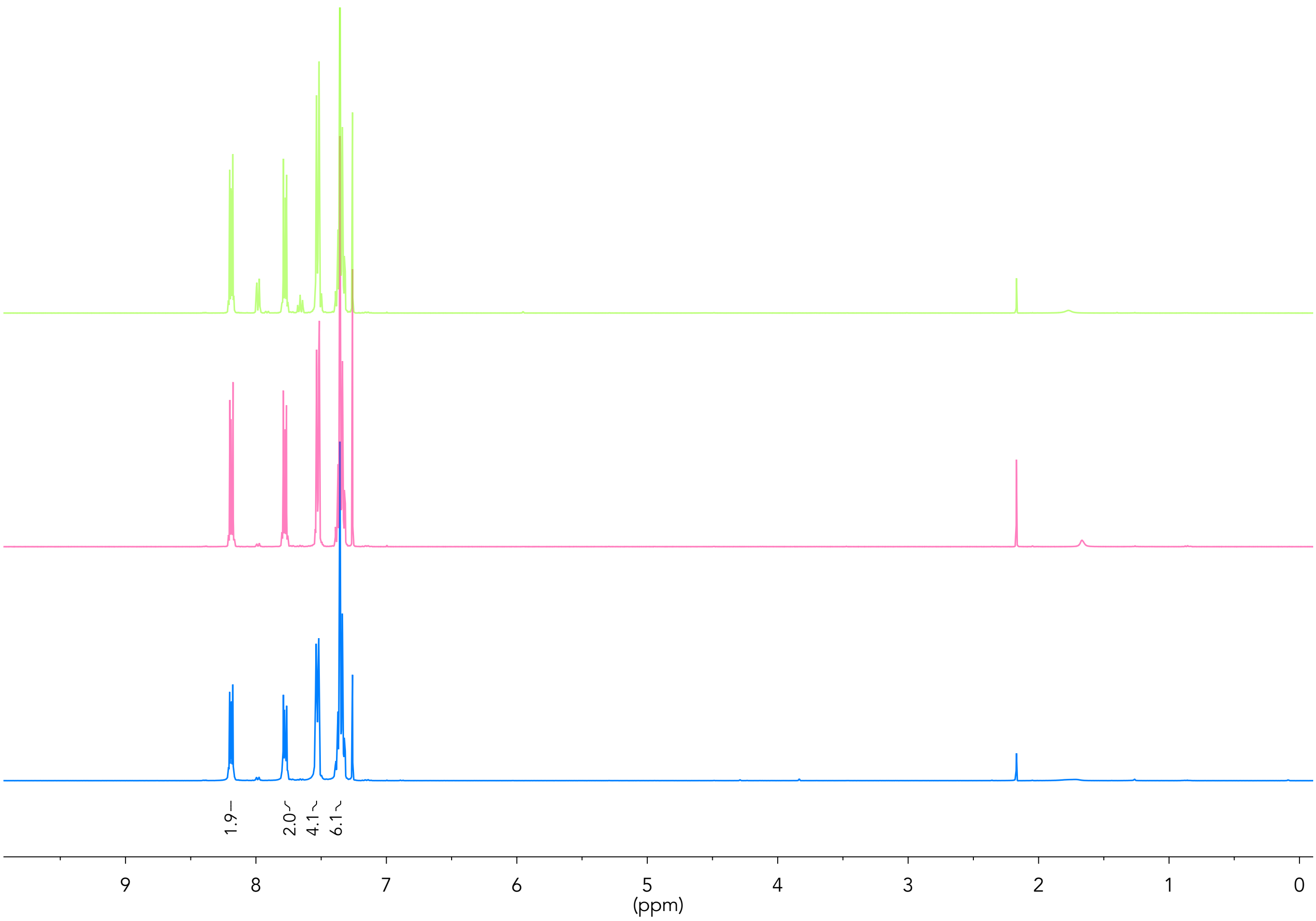

Figure SI32. ^{1}H NMR (400 MHz, DMSO-d_6) of crude reaction product between o-PDA dihydrochloride and benzil at different reaction temperatures: T_r=230 °C (Bottom), T_r=200 °C (middle), T_r=180 °C (Top).

4. Overview of number of reported quinoxalines in literature and solvents employed in their synthesis

We performed two database searches (Reaxys and Sci-Finder) on June 12th 2020. We looked for number of compounds that include the quinoxaline moiety as substructure (**Figure SI33A**). Searching for quinoxaline moiety as substructure, the Reaxys search gave a total number of 107013 hits whereas the Sci-Finder search gave a total of 323640 hits. Then, we also looked for reactions between 1,2-diketones and *o*-phenylendiamine derivatives to synthesize 2,3-disubstituted quinoxalines. We employed the search queries indicated in **Figure SI33B**. For Reaxys, a filter of single reaction step was applied and a total of 6031 hit reactions were found. For Sci-Finder, filters of single reaction step followed by considering only reaction with formation of N/O/S heterocycles were applied and 6881 hit reactions were found. The top-10 solvents and reagent/catalysts associated to these reactions are listed in **Figure SI34**.

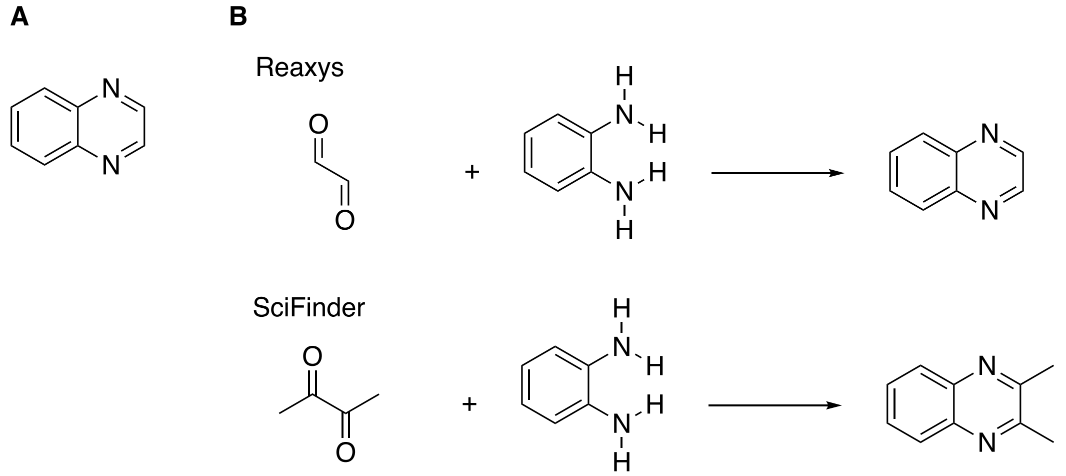

Figure SI33. **A.** Substructure employed in the search for number of reported compounds in databases. **B.** Query in the search of reactions to synthesize 2,3-disubstituted quinoxalines. For Reaxys we employed a 1,2-diketone without explicit substituents to cover as many structural analogues as possible. For Sci-Finder, all the nitrogen atoms in the structures were locked.

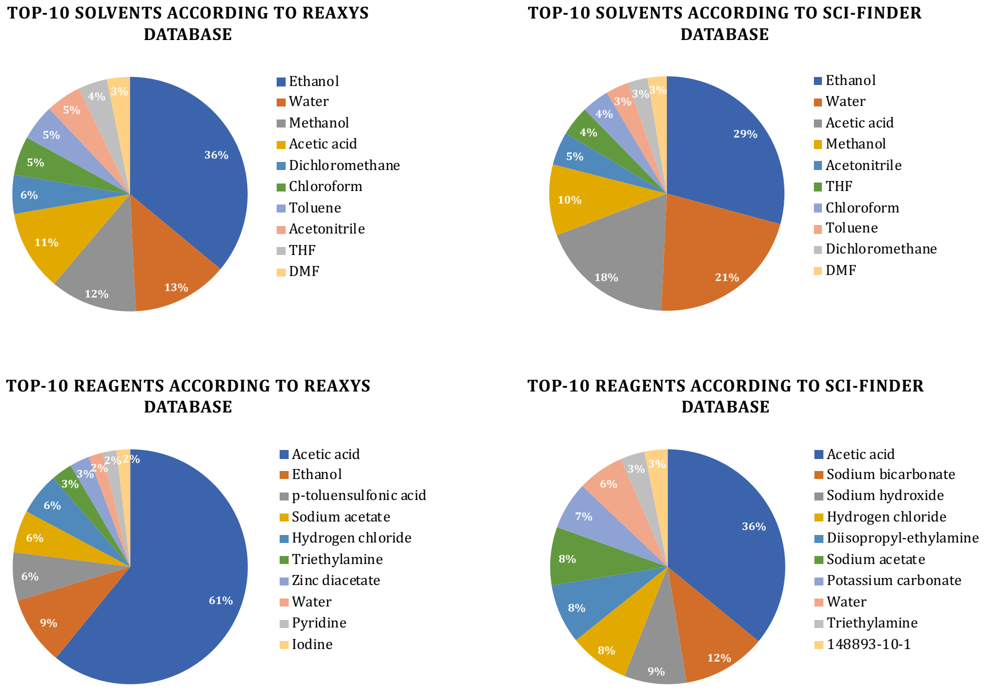

Figure SI34. Top-10 solvents and reagent obtained for the queries in Figure SI33B.

5. Computational analysis of the reaction space

The complete computational analysis was performed using Python v3.7.2. All code can be accessed in the form of jupyter notebooks via github under the following url: [UnterlassLab github page – https://github.com/UnterlassLab/Computational_Analysis_HTS_2-3-diarylquinoxalines].

5.1. Data acquisition and feature normalization

To acquire the majority of reported syntheses of the 14 hydrothermally synthesized quinoxalines, we performed a manual search of each individual quinoxaline in the commercial database Reaxys. For reasons of comparability, we have limited the search to syntheses using the same starting compounds as we have used in the HTS of the compounds. Theses searches cannot be claimed to be complete with respect to all syntheses for the respective compound using the same starting compounds ever reported, but complete with respect to all these syntheses within Reaxys at the time of the search. In sum 581 reported reactions for the 14 compounds were acquired from Reaxys. **Table SI1** summarizes the number of syntheses per individual compound, and the date of the Reaxys search. We download the details of the reported reactions as summary files for each compound in .csv format. To the extracted .csv files, we added a further row with the information of the HTS of our compounds as synthesized by method B (i.e. MW, HOAc 5%, t_R= 10 min, T_R = 230 °C). We chose to use the data of method B to represent our HTS syntheses, (i) the biquinoxalines 12-14 were only synthesized by method B (and hence method B is the one of the two methods that was used for all compounds), and (ii) since method B is still (despite using 5% HOAc as catalyst) more practical than A, for the shorter reaction time (t_R(A)= 60 min vs. t_R(B) = 10 min). An overall number of 595 reactions to be considered in the computational analysis results through 581 reactions from Reaxys plus 14 HTS.

Compound	Total amount of syntheses reported[a]	Number of Hinsberg cyclizations found and [number of HTS added manually] [b]
**1**	117	73 [1]
**2**	43	30 [1]
**3**	59	37 [1]
**4**	11	2 [1]
**5**	64	44 [1]
**6**	352	164 [1]
**7**	166	101 [1]
**8**	43	27 [1]
**9**	122	85 [1]
**10**	9	4 [1]
**11**	7	3 [1]
**12**	17	10 [1]
**13**	0	0 [1]
**14**	1	1 [1]

[a]Date and time of Reaxys search: December 1st, 2020 for compounds 1-14
[b]Date and time of Reaxys search: July 30th, 2019 for compounds 1-10 and February 17th, 2020 for compounds 11-14

Table SI1. Overview of all literature reported reactions for the individual 13 compounds found within the Reaxys database.

The .csv tables exported from Reaxys contain 41 categories of information as columns, from left to right. In **Table SI2**, we exemplarily show all categories and the entries of the first reaction, of our search of compound **3**. Note that **Table SI2** also gives comments on if, how and why we used these in expert-curating the tables for further analysis.

Entry	Category within the tables exported from Reaxys	Corresponding entry for the first result of the search for compound 3	Note regarding the category/Meaning of the category
1	Reaction ID	720073	The reaction ID is a unique identifier of the reaction in question, here the transformation of 1,2-diamino-benzene and 9,10-phenanthrenequinone to compound **3**
2	Reaction: Links to Reaxys	https://www.reaxys.com/reaxys/secured/hopinto.do?context=R&query=RX.ID=720073&database=RX&origin=ReaxysOutput&ln=	Following this link, one arrives at all current entries for the transformation of 1,2-diamino-benzene and 9,10-phenanthrenequinone to compound **3**
3	Data Count	(1 of 1)	When a single exported file contains reactions with different starting materials or products, this category shows (a of b) where a is the number of the individual reaction and b is the number of different reactions in the exported file. For the reaction in question, this category always shows (1 of 1) because all the entries refer to exactly the same reaction.
4	Number of Reaction Details	37	Give the overall number of syntheses found using of the transformation starting compounds to product. For compound **3**, our search led to 37 hits.
5	Reaction Rank	92	When one does not manually enter how the results of a reaction query should be ranked (which could be done e.g. by availability of the starting compounds), Reaxys sorts per as default setting by the “Reaxys Ranking”. The ranking algorithm is not disclosed by Reaxy, but is disclosed to be based on features such as the availability of certain details, the number of steps in the production of this product or the impact factor of the journal where the reaction is described.
6	Record Type	full reaction; has preparation	This entry gives information about the type of record that is listed here.
7	Reactant	1,2-diamino-benzene; 9,10-phenanthrenequinone	Lists the starting compounds separated by a semicolon.
8	Product	dibenzo[a,c]phenacine	Lists the chemical name of the product(s) of a transformation.
9	Bin		
10	Reaction	NC1=C(N)C=CC=C1.O=C1C(=O)C2=C(C=CC=C2)C2=C1C=CC=C2>>C1=CC=C2N=C3C(=NC2=C1)C1=CC=CC=C1C1=C3C=CC=C1	It gives the description of the reaction in SMILES code. It includes both starting materials and reaction products.
11	Reaction Details: Reaction Classification	Preparation	Classifies the report of reaction according to Reaxys classes
12	Example label		When it is available, this category shows the label of the specific section of the paper where the corresponding compound is located.
13	Example title		When it is available, this category shows the title of the section in the paper where the synthetic procedure is described (e.g. general procedure, experimental section, supporting info)
14	Full text of reaction	General procedure: reactions are carried out in a vibrating ball-mill	This entry gives the description of the synthesis in the corresponding paper

		Pulverisette 0 (Fritsch, Germany) in 4 h with an agate single ball. The reactants were introduced in equimolar ratio and in solid state (powder, flakes, etc.) under atmospheric conditions (air at room temperature and pressure). The total mass introduced in the ball-mill was 2 g, the vibration frequency was 50 Hz, and the vibration amplitude was 2.5 mm. All chemicals were purchased from Aldrich and were used without any additional treatment (the purities of the reactants are technical grade and comprise of between 95percent and 97percent for each compound).	as found there e.g. in the experimental section. Note that this field is empty for many reactions. For instance, among the 37 reports on the transformation of 1,2-diamino-benzene and 9,10-phenanthrenequinone to compound **3**, only 14 feature a text here.
15	Number of Reaction Steps	1	Gives the number of reaction steps.
16	Multi-step Scheme		Gives the reaction ID and codes for all the steps involved in a synthetic route.
17	Multi-step Details		Gives a description of starting materials and reaction conditions that correspond to each step in a synthetic route.
18	Number of Stages		If provided, gives the number of stages performed in the reaction steps (e.g. Stage 1: pre-mixture of staring material A and catalyst followed by Stage 2: addition of second starting material and heating).
19	Solid Phase		
20	Time (Reaction Details) [h]	4	Gives the reported reaction time (t_R) in hours
21	Temperature (Reaction Details) [C]	20	Gives the reported reaction temperature (T_R) in °C
22	Pressure (Reaction Details) [Torr]		Gives the reported reaction pressure (p_R) in Torr; very seldomly reported
23	pH-Value (Reaction Details)		Gives the reaction pH; very seldomly reported
24	Other Conditions	Neat (no solvent); Solid state	Gives additional information on the reaction conditions. We saw labels such as "green chemistry", or "reflux", "ionic liquid", or "microwave irradiation". This category often does not contain any text (empty field).
25	Reaction Type		Typically, empty field; would potentially contain information on the type of reaction (e.g. condensation, cross coupling, oxidation, etc)
26	Subject Studied		Typically, empty field; we have seen entries such as "mechanism". It is only seldomly filled.
27	Prototype Reaction		Typically, empty field; we have seen entries such as "reagent/catalyst"; For instance, going to the original literature of syntheses that were labeled as "reagent/catalyst", the corresponding articles did not report the development of a new reagent or catalyst, but e.g. use very conventional catalysts (e.g. zeolite Y).
28	Named Reaction		It would potential give the name of the transformation if the transformation was a name reaction

29	Type of reaction description (Reaction Details)		Often empty; sometimes features e.g. “experimental part” so we conclude that the entries specifies where the reaction details were found. In the case of the example synthesis of compound **3**[1] the synthesis description featured in entry 14 (Fulltext of reaction) is displayed as “general procedure”
30	Location	Experimental part	Gives the location of the synthesis description in the corresponding article; we found entries such as “experimental part” or “scheme or table”
31	Comment (Reaction Details)		Typically empty
32	Product	dibenzo[a,c]phenacine	Specifies the reaction product; identical with entry 8
33	Yield	> 99 percent	Gives reaction yield
34	Yield (numerical)	99	Gives the reaction yield as number (excluding e.g. “<” and without the unit %); useful for computational purposes.
35	Yield (optical)		We found this entry to be typically empty in the tables for our compounds, which is of course due to the fact that they are non-chiral. Typically, this entry would give optical yield given as % ee.
36	Stage Reactant		It usually only shows information for procedures that need more than one stage in a single reaction step. It shows the staring materials that match with the query search.
37	Reagent		Technically this entry would give reagents (as per IUPAC definition: “A substance that is consumed in the course of a chemical reaction. It is sometimes known, especially in the older literature, as a reagent, but this term is better used in a more specialized sense as a test substance that is added to a system in order to bring about a reaction or to see whether a reaction occurs (e.g. an analytical reagent))[4].However, databases use this term differently, and e.g. in SciFinder, a reagent will be used to designate compounds that contribute non-carbon atoms to the product.[2] In the tables downloaded from Reaxys for compounds 1-14 we found this entry to typically contain the employed catalysts or promotors. Therefore, we have merged the entries “reagent” and “catalyst” for our further data analysis.
38	Catalyst		Typically empty field, even when for synthesis in articles entitled “[...]Synthesis of quinoxalines using [...] as catalyst”; Therefore, we merged the contents of this entry with the the “reagent” entry.
39	Solvent (Reaction Details)		Gives the solvent that was used
40	References	Article; Carlier, Leslie; Baron, Michel; Chamayou, Alain; Couarraze, Guy;	Details the literature reference from which the synthesis was extracted

		Tetrahedron Letters; vol. 52; 36; (2011); p. 4686 - 4689;	
41	Link to Reaxys	https://www.reaxys.com/reaxys/secured/hopinto.do?context=C&query=CNR.CNR=9078488&database=RX&origin=ReaxysOutput&ln=	Gives the link to the precise synthesis entry in Reaxys; useful for manually assessing the entry

Table SI2. Overview of all categories offered within a synthesis table exported from Reaxys, at the example of compound **3** and the first entry within the search result table. We limited our searches for all compounds to the same starting compounds used, which in the case of compound 3 are 1,2-diamino-benzene and 9,10-phenanthrenequinone. Categories that we used in our data analysis (entries 20, 21, 34, 37+38(merged)) are highlighted by blue background.

As becomes clear from **Table SI2**, Reaxys theoretically offers at least 41 different data categories, of which potentially approx. 20 are useful as reaction input or output parameters, while others are useful for e.g. manually checking the respective Reaxys database entry (links) or reading the corresponding reference. Entries that would be useful reaction parameters for use in our computational analysis include e.g. pressure and pH-values. Unfortunately, the majority of them are only poorly reported. Therefore, we included only temperature, time, yield(numerical), as well as solvent, regent, and catalysts for our data analysis. All other parameters were discarded for the following analysis. We decided to merge the categories reagent and catalyst, as (i) the category reagent typically included what is actually catalysts, and (ii) since we were in the following interested in the harmfulness of a reaction, we wanted to assess all chemicals present in a reaction mixture. The assessment of the hazard of solvent and reagent+catalyst (in our analysis denoted collectively as catalyst) is described in the following paragraph. All unfiltered synthesis tables as extracted from Reaxys are available at our github reopository https://github.com/UnterlassLab/Computational_Analysis_HTS_2-3-diarylquinoxalines. Next, we filtered all 595 reactions for those not reporting any values for yield, temperature or time, and removed these entries, resulting in 323 final reactions. Of these final 323 reactions, we removed all categories not used in the further analysis, and as mentioned already, merged the categories reagent and catalyst. Note that these 323 final reactions were used to generate the spider plot of all reaction parameters displayed in the manuscript (**Fig 1B**), whereas different numbers of reactions were used to generate the histograms of how each of the parameters is distributed across all syntheses. E.g. within the 595 reactions, n= 538 entries had an entry for reaction time t_R (yet were not necessarily complete with respect to the set of 6 parameters investigated here). To use the biggest possible amount of data for the analysis of each parameter, we used reactions that featured the corresponding parameter for generating the histograms displayed in the manuscript in Fig.1B. The corresponding numbers of used values are: Solvent n=452; Catalyst: *n*=484; Temperature: *n*=479; Time *n*=538; Yield: *n*=513.

Numerical parameters such as temperature [°C], time [h] and numerical yield (i.e. display of e.g. a yield of 70% as 0.7) could be directly used for the analysis, while the features solvent and catalyst (merged with reagent) first needed to be quantified. We quantified the hazardousness-of the 25 different used solvents and solvent mixtures according to Bryne.[3] Each solvent was assigned a number between 1 [recommended] and 6 [highly hazardous]. For reactions that used a mixture of different solvents, the most hazardous solvent was used. Catalyst toxicity was assessed using the Globally Harmonized System (GHS). Therefore, we manually annotated all 122 catalysts found in the reported literature with their respective hazard statements (H-codes).

For the assessment of toxicity through the GHS, we used the PubChem online GHS resource (https://pubchem.ncbi.nlm.nih.gov/ghs/). The H-codes correspond to a Hazard statement (e.g. H200 (H-code) corresponds to the Hazard statement "unstable explosive"), a Hazard class (here "explosives"), and a hazard category (here "unstable explosives"). Furthermore, every H-code comes with a set of precautionary statements (P-codes), which are recommendations for preventing these dangers, responding to them shall they arise, store the compound safely, and dispose of the compounds. As the P-codes are a direct consequence of the H-codes, we did not consider them for our computational analysis, but used the H-codes only. Below, an exemplary entry of an H-code with associated statement and categorization as displayed in the PubChem resource is shown:

Code	Hazard Statements	Hazard Class	Category	Pictogram	Signal Word	Precautionary Statements P-Codes			
						Prevention	Response	Storage	Disposal
H200	Unstable Explosive	Explosives	Unstable Explosive	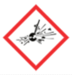	Danger	P201, P202, P281	P372, P373, P380	P401	P501

The H-codes belong to a category (fourth column in the above table), which can be a category, a type, or a division (in the table one finds entries such as "Category 1", "Category 2", "Div 1.5", "Type A", "Type B", "Type C,D"). These

categorizations can be used to scale the hazardousness, which we did in the following for all catalysts used in the synthesis of the 14 quinoxalines according to our Reaxys search results.

Each catalyst can have between zero and several H-codes. For reactions that used a mixture of several catalysts, we assigned the complete set of all found H-codes to that reaction. The hazardousness for each H-code was scaled according to its division, type or category, so that the most severe H-code within one group was assigned a scaled hazardousness (H_S) of 1, and the least severe H-code within the same group was assigned a value of 1/[number of H-codes within group]. Finally, the overall hazardousness (H) for each reaction was calculated by using the sum over all individual H_S assigned to that reaction. We supply tables of the hazardousness of all compounds as supporting file.

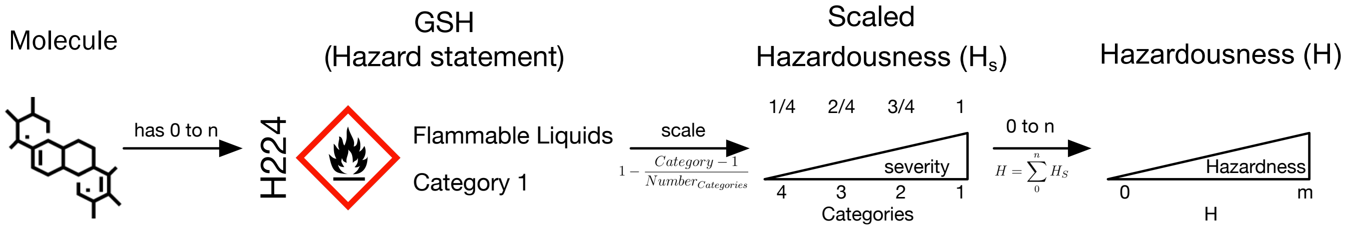

Figure SI35. Scheme showing how catalyst hazardousness was calculated for each reaction.

5.2. Individual spider plots for each compound

To create meaningful spider plots, we normalized all parameters between zero and one, i.e., zero corresponding the minimum of all reported values for this parameter and one for the maximum respectively. This was necessary as the relative range between the individual features was quite broad, e.g. temperature [20-230] compared to solvent toxicity [1-6].

$$x_{scaled} = \frac{x - F_{min}}{F_{max} - F_{min}}$$

In the following, spider plots for each of the 14 compounds considering all 5 scaled reaction parameters are shown. Note that n represents the number of syntheses reported, which ranges between n=1 (compound 14, conditions reported in this work) and n=89 (compound 6, quite exploited in the literature according to our Reaxys search).

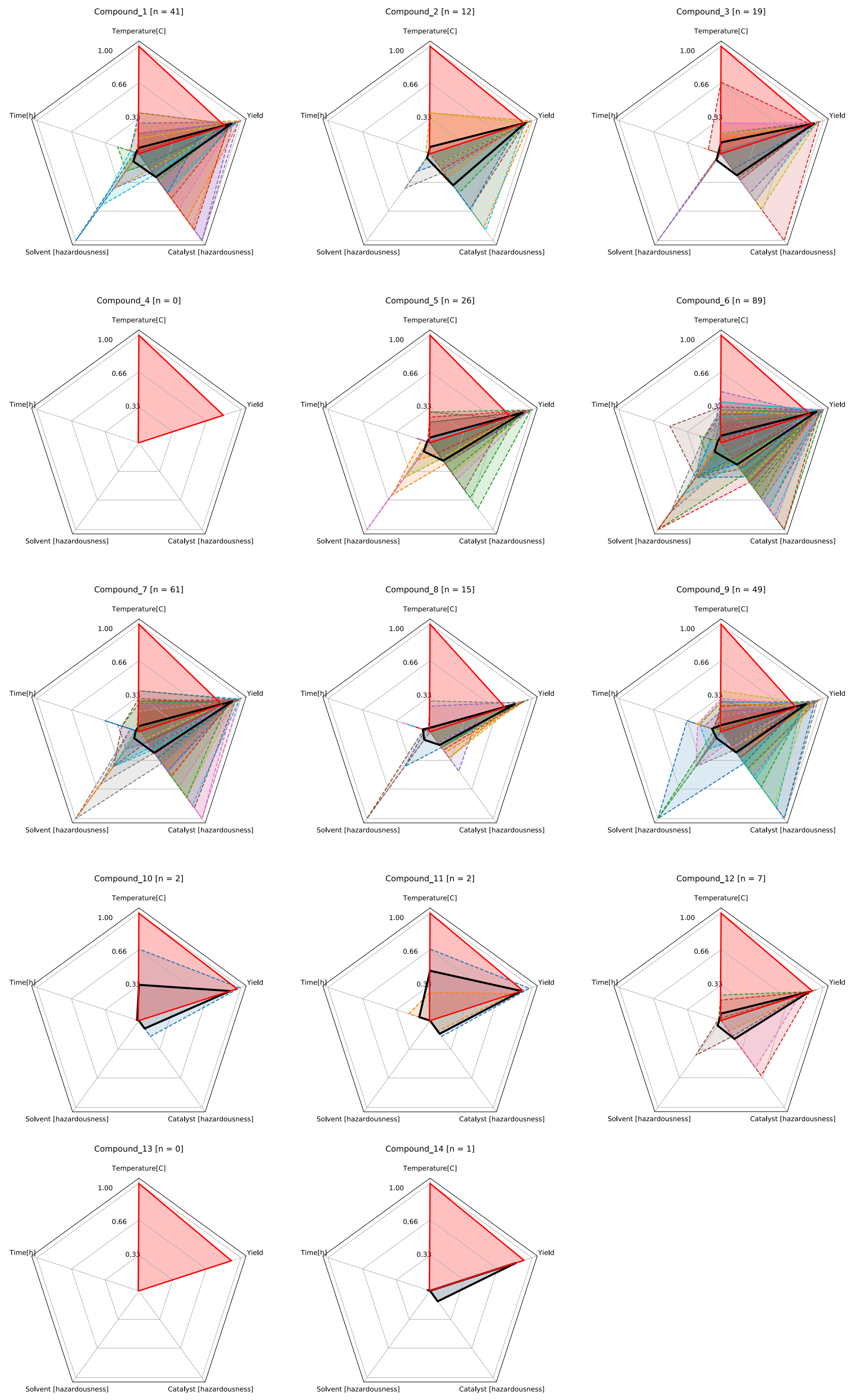

Figure SI36. Spiderplots showing the reaction space for each of the 14 compounds individually. The thick red line corresponds to the hydrothermal reaction. Dashed colored lines represent literature known reaction. The thick black line shows the mean results over all literature reactions.

5.3. Dimensionality reduction techniques

We performed both PCA (Principal component analysis, Figure 1C and t-SNE (t-distributed Stochastic Neighbor Embedding, Figure S35) to reduce the number of dimensions from 5 to 2. Essentially, the main difference between these two-dimension reduction techniques is that PCA works by reducing the dimension in a way that

it separates points as far as possible (keeps variance), while t-SNE works by grouping points as close as possible (keeps local similarities). For t-SNE we used the following parameters: perplexity=45.0, learning_rate=200.0. Prior to dimension reduction, all features were scaled by removing the mean and scaling to unit variance.

$$x_{scaled} = \frac{(x - \mu)}{\sigma}$$

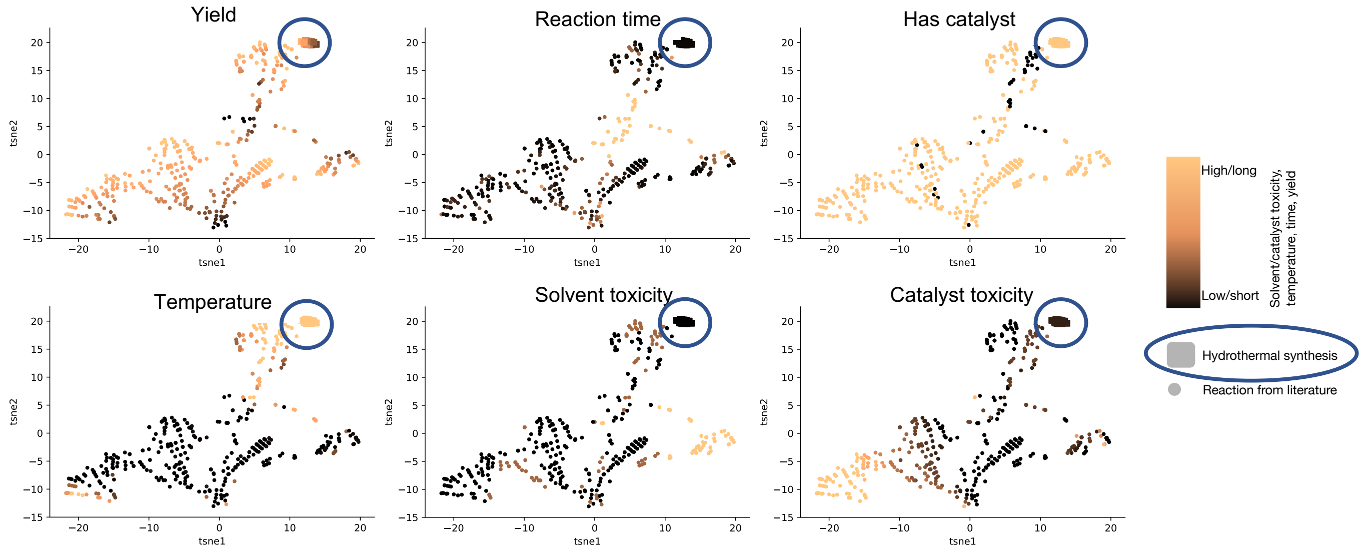

Figure SI37. t-SNE analysis with overlay of the individual features. Squares correspond to hydrothermal reaction, while point to literature reported. The color intensity reflects the extend of the individual features, e.g., high temperature, high yield or long reaction time.

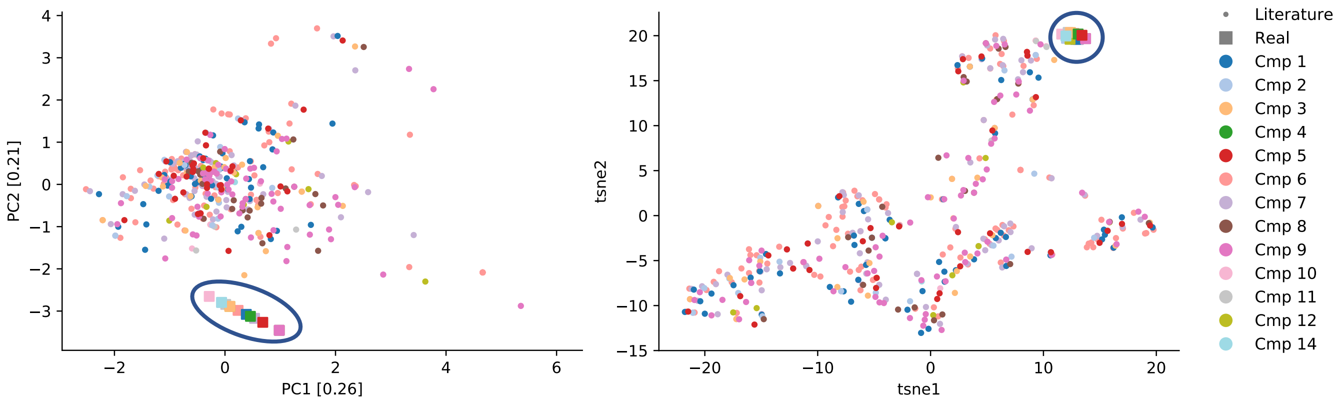

Figure SI38. Both the PCA (left) as well as the t-SNE (right) analysis reveal no intimate relationship between compound and reaction condition specificity. Reactions are clustered according the specific reaction types, e.g., all hydrothermal reactions (inside blue circles) are located almost at the same spots as they are identical in all reaction conditions with exception of their yields.

5.4. K-means cluster analysis

We identified clusters of reaction conditions using k-means clustering, with k = 8 (Manuscript Figure 1E). This number k refers to the number of centroids you align your dataset to. Each data point in the cluster is then assigned to exactly one centroid through reducing the in-cluster sum of squares. Each of the 8 identified clusters showed an overall unique fingerprint of reaction conditions (Figure S37).

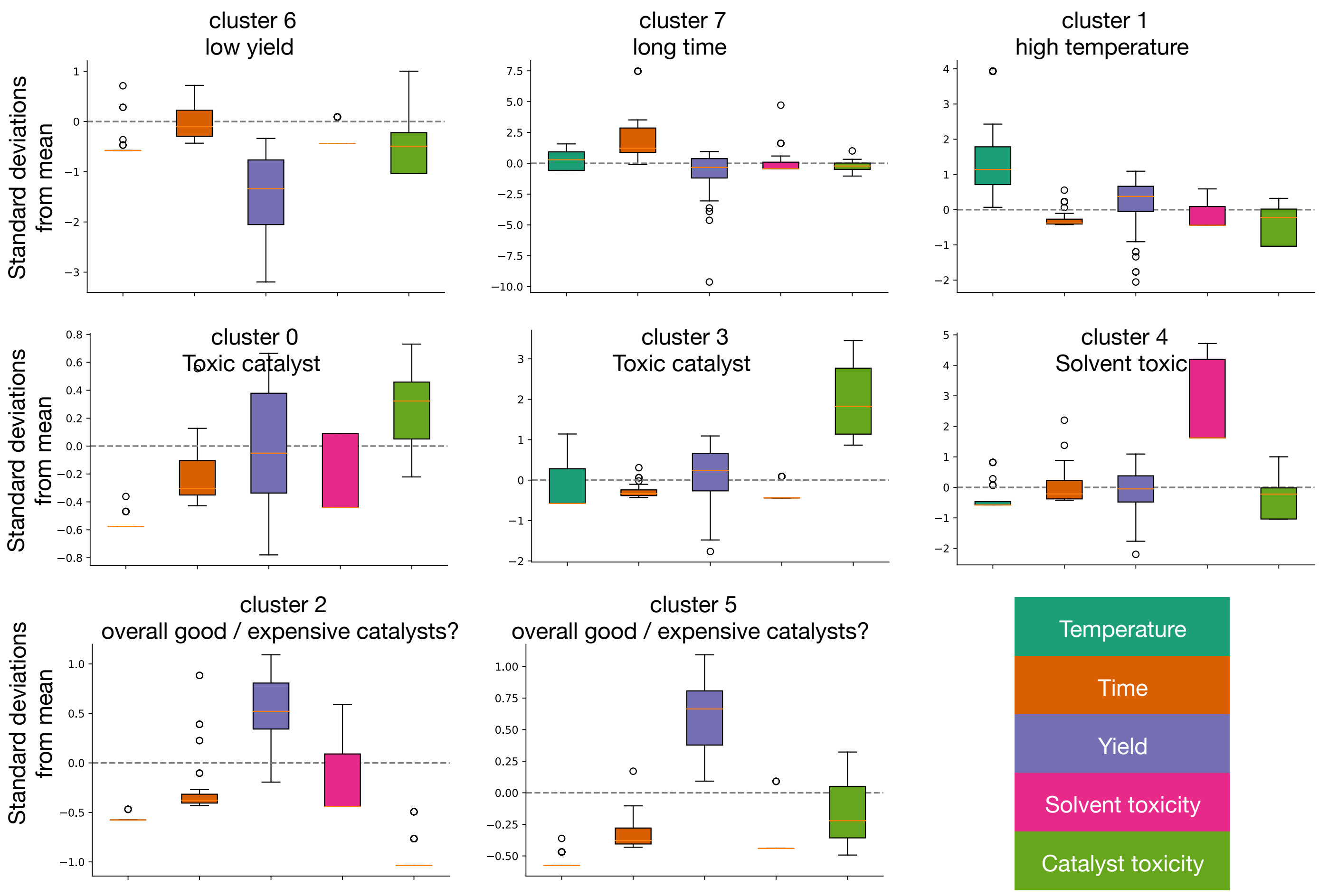

Figure SI39. Boxplots showing the individual reaction parameter fingerprints for the 8 different clusters. Roughly the clusters can be categorized into: (i) low yield, (ii) long reaction time, (iii) high temperature, (iv) toxic catalyst, (v) toxic solvent and (vi) use of optimized catalyst. Examples of catalysts that belong to cluster 2 and 5 are: Zirconia supported tungtophosphoric acid, amberlyst-15, titanium (IV) oxide, lanthanum (III) triflate, propylsulfonic acid functionalized nanozeolite clinoptilolite.

6. Fluorescence measurements and fluorescence microscopy experiments

6.1. Fluorescence measurements of quinoxalines 1-14

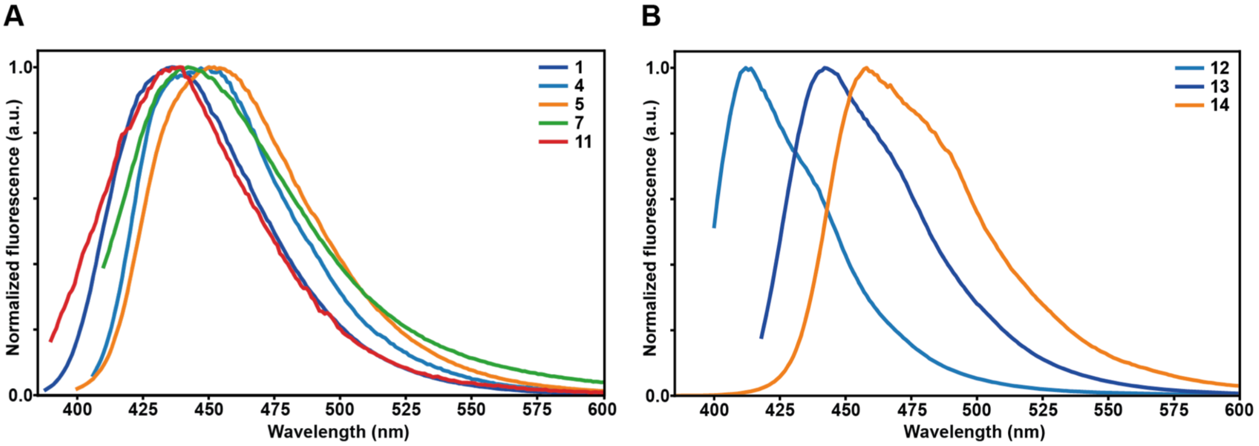

Figure SI40. Normalized emission spectra of selected quinoxalines (A) and biquinoxalines (B). Measurements were done with solution 5 μM in $CHCl_3$ (λ^{exc} =380 nm)

6.2. Fluorescence microscopy

The corresponding cell lines were maintained in 96 well plates (15000 cells/well). The cells were maintained under 5% CO_2 atmosphere at 37 °C. For cell staining experiments, 100 mM stock solutions of quinoxalines in DMSO were diluted to 10 mM by hand and cells were treated with diluted solutions of quinoxalines to get different final concentrations: 45, 15, 5 and 1.5 μM. Cells were incubated for 45 minutes at 37 °C under 5% CO_2 atmosphere and lived-imaged using an Opera Phenix high-content fluorescence microscope (PerkinElmer) with 40X objective.

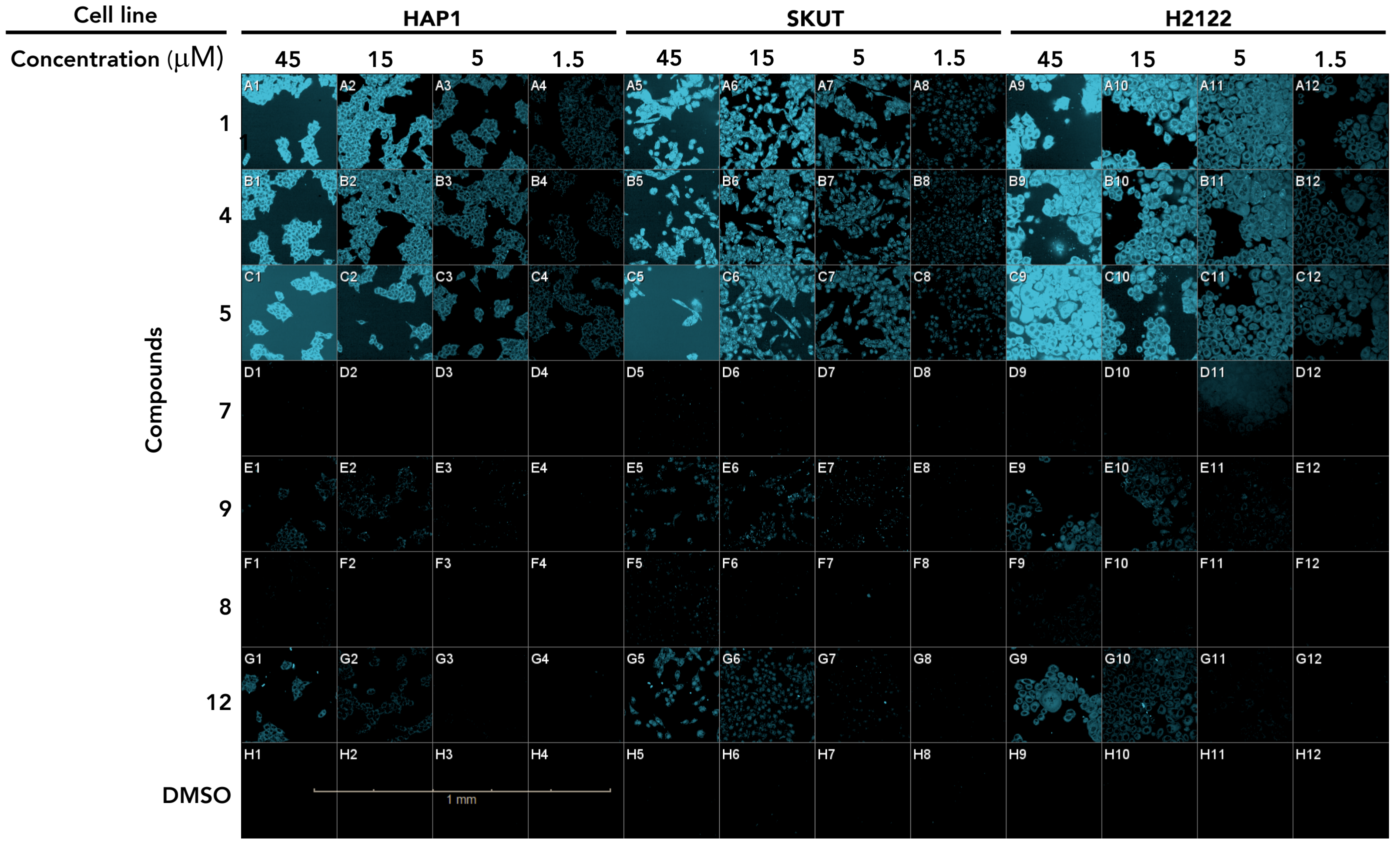

Figure SI41. Fluorescence microscopy images after treating the corresponding cell lines with solutions of the indicated quinoxalines.

References

[1] L. Carlier, M. Baron, A. Chamayou, G. Couarraze, *Tetrahedron Lett.* **2011**, *52*, 4686–4689.

[2] T. Engel, J. Gasteiger, *Chemoinformatics: Basic Concepts and Methods*, Wiley, **2018**.

[3] F. P. Byrne, S. Jin, G. Paggiola, T. H. M. Petchey, J. H. Clark, T. J. Farmer, A. J. Hunt, C. Robert McElroy, J. Sherwood, *Sustain. Chem. Process.* **2016**, *4*, 1–24.

[4] https://goldbook.iupac.org/terms/view/R05163
